# A motion capture protocol for the kinematic analysis of transfemoral and transtibial sprinters

**DOI:** 10.3389/fbioe.2025.1655295

**Published:** 2025-11-04

**Authors:** Roberto Di Marco, Samira G. Breban, Giuseppe Zullo, Francesca Gariboldi, Mattia Scapinello, Gian Luca Migliore, Nicola Petrone, Andrea G. Cutti

**Affiliations:** ^1^ Department of Engineering for Innovation Medicine, University of Verona, Verona, Italy; ^2^ Centro Protesi, INAIL, Budrio, Italy; ^3^ Department of Industrial Engineering, University of Padova, Padova, Italy

**Keywords:** paralympic running, biomechanical model, lower-limb amputation, open-access software, standardized procedure

## Abstract

Optimizing performance and safety in sprinters with lower-limb amputation requires standardized methods. This study presents a novel marker-based motion capture protocol to define local coordinate systems and Cardan sequences for *in-vivo* analysis of running biomechanics in athletes with transfemoral (TF) and transtibial (TT) amputation. The protocol provides detailed definitions and shares computational codes, supporting prosthetists and coaches in optimizing prosthetic setups. Moreover, integrating *in-vivo* biomechanics data into *in-vitro* and *in silico* experiments could lead to safer, more effective prosthetic designs. The methodology was tested involving two Paralympic gold medallists (one TF, one TT). To support global adoption and broad validation, all necessary computational tools, including kinematic calculation codes and model configuration files, are openly provided. These resources enable researchers to apply the protocol to various prosthetic setups and further test its applicability. By fostering global collaboration, this work lays the foundation for analysing Paralympic sprinting, optimizing athletic performance, improving prosthetic design, and advancing Paralympic sports biomechanics.

## 1 Introduction

Public interest in Paralympic sports is growing as recently demonstrated in Paris for the 2024 Games. This growth is driven by global events and the influence of social media, which are attracting more people with disabilities to participate in sports at highly competitive levels of athletes (Bouzas et al., 2021; Bragaru et al., 2011).

When considering persons with transfemoral (PTFA) or transtibial amputation (PTTA), athletic performances can be influenced by the components adopted in the athlete’s Running Specific Prosthesis (RSP) (Oudenhoven et al., 2017; Sakata et al., 2020) as well as by their set-up, also referred to as “prosthetic alignment” (Fletcher et al., 2021; Migliore et al., 2021). Both topics are actively investigated to allow Paralympic athletes safely compete at the highest level, delivering maximal performance without compromising their comfort and body integrity (Hadj-Moussa et al., 2022; Kent and Franklyn-Miller, 2011). Research methods include *in-vivo* quantitative collection of motion, followed by biomechanical data analysis (Alcantara et al., 2022; 2021; Day et al., 2021; Hobara et al., 2014; 2013; Makimoto et al., 2017; Nagahara et al., 2018; Taboga et al., 2020), component bench tests (Beck et al., 2016; Cutti et al., 2023; Dickinson et al., 2023; Gariboldi et al., 2023b; Gariboldi et al., 2023a; Gariboldi et al., 2022), and *in silico* simulations (Barattini et al., 2023; Murai et al., 2018; Rigney et al., 2015).

The biomechanical analysis of Paralympic athlete performances is crucial to deepen the understanding of both the athlete-prosthesis interaction, and the link between prosthesis set-up and performance. In the past decades, attention was paid to both kinematics and kinetics analyses. For sprinting, kinetics analyses call for expensive experimental set-up with force plates installed on track (Hobara et al., 2014; Hobara et al., 2013; Makimoto et al., 2017; Nagahara et al., 2018). These high costs have led to the adoption of the more cost-effective instrumented treadmill (Alcantara et al., 2022; Alcantara et al., 2021; Day et al., 2021; Taboga et al., 2020), allowing for data collection from many repetitions under highly controlled environments (Morin and Sève, 2011). However, treadmills have the drawback of not reproducing nor the top flying speeds nor the sprinting acceleration, which is a key element in understanding how athletes achieve high velocity (Maćkała et al., 2015), as well as altering the interaction with floor (tartan for sprinting tracks), aerodynamics and the perception of the surrounding space. Moreover, it imposes a single speed for both legs, while the body of a person using a prosthesis is clearly asymmetrical, with net braking impulses on the sound side and net propulsive impulse on the affected side (Breban et al., 2022).

Regarding kinematic analysis using marker-based stereophotogrammetry, most of the published studies focus on walking analyses (Hadj-Moussa et al., 2022; Kent and Franklyn-Miller, 2011). However, the approaches used in those studies cannot be adopted in sports assessments due to differences in the prostheses, particularly the running prosthetic feet. When defining marker-based kinematic models, some studies base the marker positioning on the prosthetic side on similarities with the sound limb, others associate markers to mechanical parts, and some use mixed approaches (Kent and Franklyn-Miller, 2011). Thus, no established motion analysis protocols (Kontaxis et al., 2009) exists for studying the kinematics of athletes with lower limb amputation in sports applications, resulting in data misinterpretation across studies and hampering the method’s use in assessing prosthetic function (Sawers and Hahn, 2010). Moreover, *in vivo* data collection is a key factor for setting up mechanical bench tests capable to reproduce real-world conditions to assess the functional characteristics of running specific components and sockets (stiffness) and their structural characteristics (ultimate strength and lifecycle). Component failure can indeed lead to prosthesis malfunction and potential athletes’ injuries (Gariboldi et al., 2022). Unfortunately, the lack of established motion analysis protocols limits the definition of standardized bench testing protocols, limiting the validity of the results across laboratories (Gariboldi et al., 2025a; Gariboldi et al., 2025b; Gariboldi et al., 2022).

In this framework, this study had three aims: 1) to propose a new method to concurrently assess *in-vivo* kinematics of running in athletes with TF and TT amputation, and inform bench test settings (e.g., relative position and orientation of prosthetic components, magnitude and directions of forces to be applied) of their prosthetic components; 2) to distribute to the research community the software needed to calculate the reference frames and joint angles proposed herein, ensuring transparency and reproducibility in biomechanical assessments; 3) to show two paradigmatic case studies, involving two Paralympic gold medallist with transfemoral (TF) and transtibial (TT) amputation.

## 2 Materials and methods

The following sections presents the nomenclature, the segments to be tracked, the marker positioning, the Local Coordinate System (CS) definitions and the Cardan sequences to be used to estimate joint kinematics.

Local Coordinate Systems (CS) were associated with each anatomical segment and prosthetic component, defining local pose matrices (position and orientation). Relative motion between segments or mechanical parts were computed starting from local pose matrices.

### 2.1 Terms, definitions, and notations

#### 2.1.1 Prosthesis

A Running Specific Prosthesis (RSP) consists of a socket (the part that accommodates the residual limb), a prosthetic knee (in case of TF amputation) and a prosthetic foot, which for sports applications is hereinafter called Running Prosthetic Foot (RPF) as in (Petrone et al., 2020). Their relative position and orientation are set and maintained through “adapters” (clamps, hinge, pyramid or sliding joints with setscrews), and “pylons” (tubes of various length). Supplementary Figure 1 shows the typical configurations (assembled and exploded) of a RSP limb for TF (top side) and TT (bottom side) amputations.

Since RSP are modular systems, each component has a proximal and/or distal interface depending on its position in the serial connection. Among the connecting interfaces, two are particularly relevant for the prosthesis set-up, and, thus, for the definition of the motion analysis protocol: (i) the interface between the socket and its distal component is referred to as “*socket-clamp*”; (ii) the proximal interface of the prosthetic foot is named “*foot-clamp*”. In case of TT prosthetic limb, the socket-clamp and foot-clamp degenerate into a single foot-clamp.

The most used prosthetic knee in RSP is a mechanical monocentric knee joint (Ottobock 3S80, Ottobock GmbH, Duderstadt, Germany). It enables to adjust flexion and extension damping separately to ensure optimal control of flexion angles and extension during the swing phase of running. For elite running and sprinting, this is the only prosthetic knee used at present.

**FIGURE 1:**
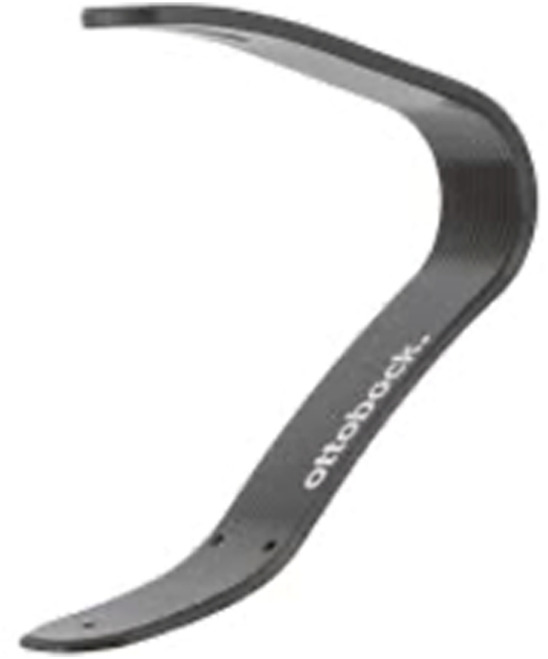
Running prosthetic foot 1E91, ottobock (Germany). https://www.ottobock.com/en-ex/product/1E91-60984.

**FIGURE 2:**
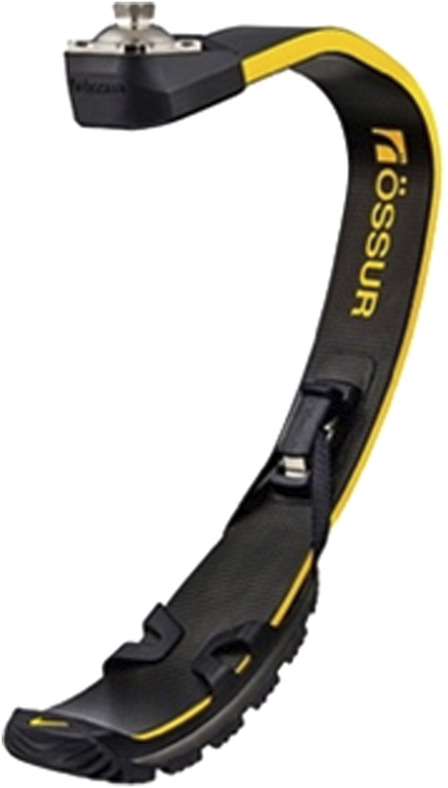
Running prosthetic foot Cheetah^®^ xceed, ossur (Iceland). https://www.ossur.com/en-za/prosthetics/feet/cheetah-xceed.

**FIGURE 3:**
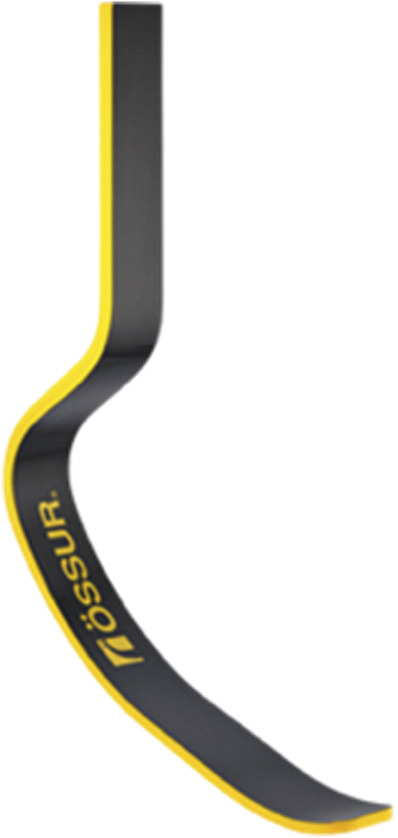
Running prosthetic foot Cheetah^®^ xtreme, ossur (Iceland). https://www.ossur.com/en-gb/prosthetics/feet/cheetah-xtreme.

**FIGURE 4:**
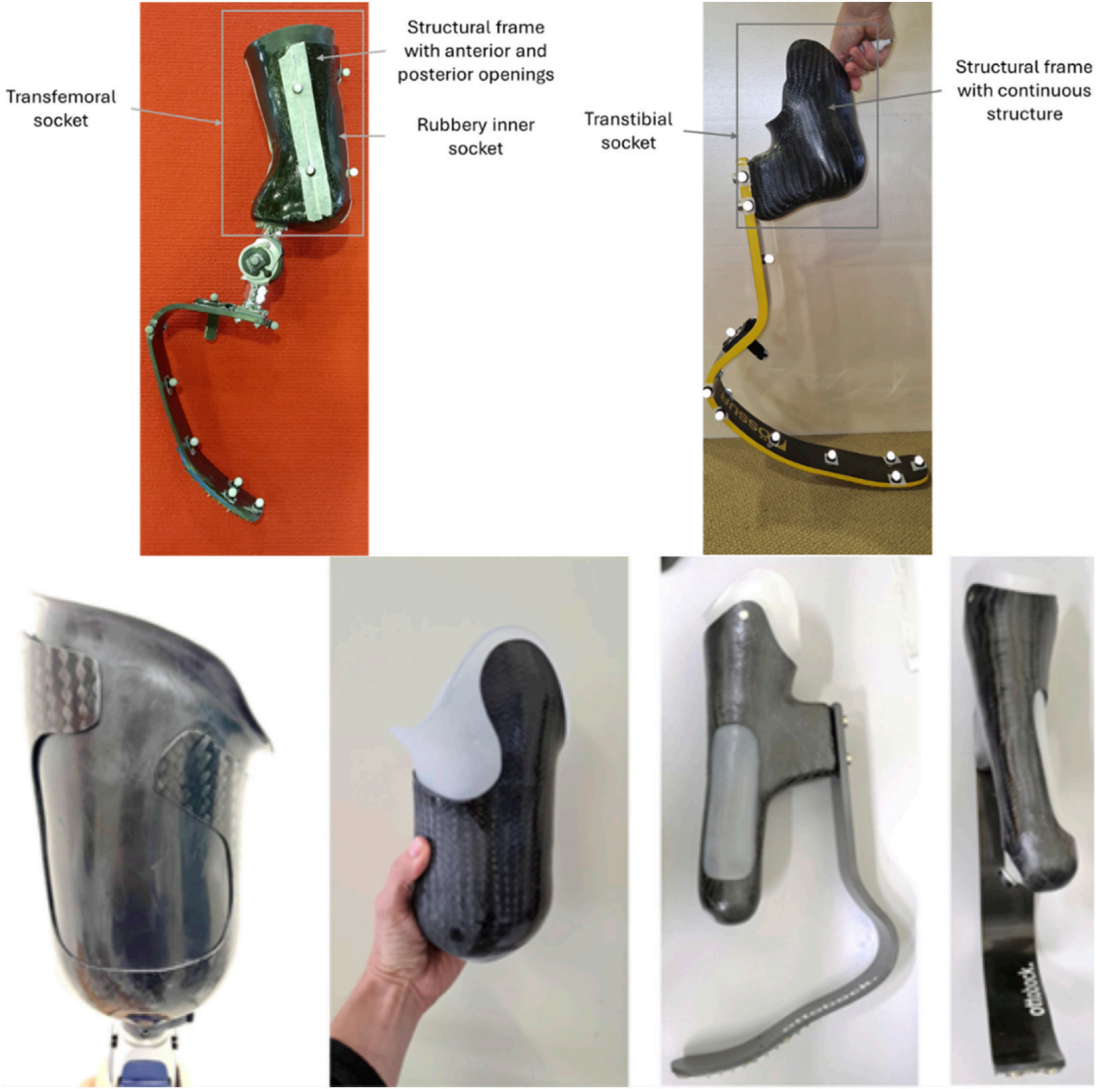
Flexible sockets for people with transfemoral (two leftmost pictures) and transtibial (two rightmost pictures) amputations.

Although RPF shape may vary depending on the manufacturer, RPF presents either a C-shape or a J-shape (Figures 1–4). Theoretically, both shapes can be mounted on TT and TF RSP. However, since C-shapes are intended to be positioned under the socket, requiring sufficient height from the ground, they are mostly included in TF RSP. On the contrary, J-shapes are intended to be positioned posteriorly, in correspondence to the socket posterior box, which makes them ideal for direct connection to TT sockets. This is also the typical configuration for elite Paralympic athletes, and it will be the one we will refer to herein.

#### 2.1.2 Coordinate systems

All CSs are defined by a right-handed orthonormal xyz triad (with the x -axis pointing forward and the y -axis being anti-parallel to gravity, having the subject in the standing posture) and are centred in an origin O . Each CS, including the global reference and those associated with body parts or mechanical segments, is identified by a subscript label in capital letters. The fixed global reference system is ${CS}_{G}$ , with the floor laying on the $\left(x_{G},z_{G}\right)$ -plane, with $x_{G}$ aligned with the running direction. For bony and mechanical segments, two capital letters abbreviate the part name and, when relevant, a prefix is added to indicate the body side: either <s> equal to L for the left, or R for the right side. For example: ${CS}_{PL}$ stands for the pelvis (PL) local CS; the CS for thigh (TH) segment would be ${CS}_{<s>TH}$ , with ${CS}_{RTH}$ referring to the right and ${CS}_{LTH}$ referring to the left thigh. Coordinate axes for local CSs are defined according to the recommendations of the International Society of Biomechanics–ISB (Wu and Cavanagh, 1995): i.e., x and y -axes define the quasi-sagittal plane, with x pointing forward (hereinafter drawn in red), y pointing upward (drawn in green), and the *z*-axis is orthonormal to the quasi-sagittal plane and points rightward (drawn in blue).

Human or mechanical joints are instead labelled with their names’ lowercase first letter (e.g., cervical is c ), with a prefix <s> being used to identify the body side (i.e., l for the left and r for the right side): e.g., lh stands for the left hip joint. When dealing with a joint modelled with the junction between a bony segment as distal body and a distal segment being a mechanical part of the prosthetic limb, a tf or tt tag is used as suffix: e.g., left hip for a PTFA with a socket on the residual left thigh is lhtf .

Points (in uppercase), vectors (in lowercase) and matrices (uppercase bold text) are labelled with a left superscript to address the CS they are defined into, and a right subscript to identify the CS they are referred to. For example:

• PG is the point P, whose coordinates are given in ${CS}_{G}$ (the left superscript is omitted when dealing with ${CS}_{G}$ only);
• OPLG is the origin of the pelvis coordinate system ${CS}_{PL}$ , whose coordinates are given in ${CS}_{G}$ ;
• x^LTHG is x -axis unit vector of the left thigh coordinate system ${CS}_{LTH}$ , whose components are given in ${CS}_{G}$ ;
• TLTHG is the 4 x 4 homogeneous transformation that moves points P defined in ${CS}_{LTH}$ coordinates to the ${CS}_{G}$ :

 GP1=TLTHG  LTHP1;

• RLTHG is the 3 x 3 matrix that rotates vectors $\vec{v}$ from the ${CS}_{LTH}$ to the ${CS}_{G}$ :

v→G=RLTHGv→LTH.

### 2.2 Segments, landmarks, markers, coordinate systems

This section and the Supplementary Material present the definition of each segment, together with its landmarks and the relevant markers used for their tracking (Cappello et al., 1997), and the CS.

Sound-side segments definitions follow the ISB recommendations (Wu et al., 2002), and include head, trunk, pelvis, thigh, shank, and foot. The upper limbs are also included through a simplified two-segment description, one for upper arm and one for the forearm, considering that their motion takes place mostly on the body quasi-sagittal plane during running, with the elbow not extended (Hamner et al., 2010; van Oeveren et al., 2024). Since these definitions are based on known literature, they are provided in the Supplementary Section 2, while those for the prosthetic side are included herein.

Prosthetic side segments include socket, prosthetic knee, and RPF, which is assumed to be formed by two sub-segments, the proximal portion (solid with the foot-clamp) and the distal tip portion (the distal-most 5 cm of the running blade). The relative motion of these two sub-segments accounts for the RPF large deformations during running stance.

**FIGURE 5:**
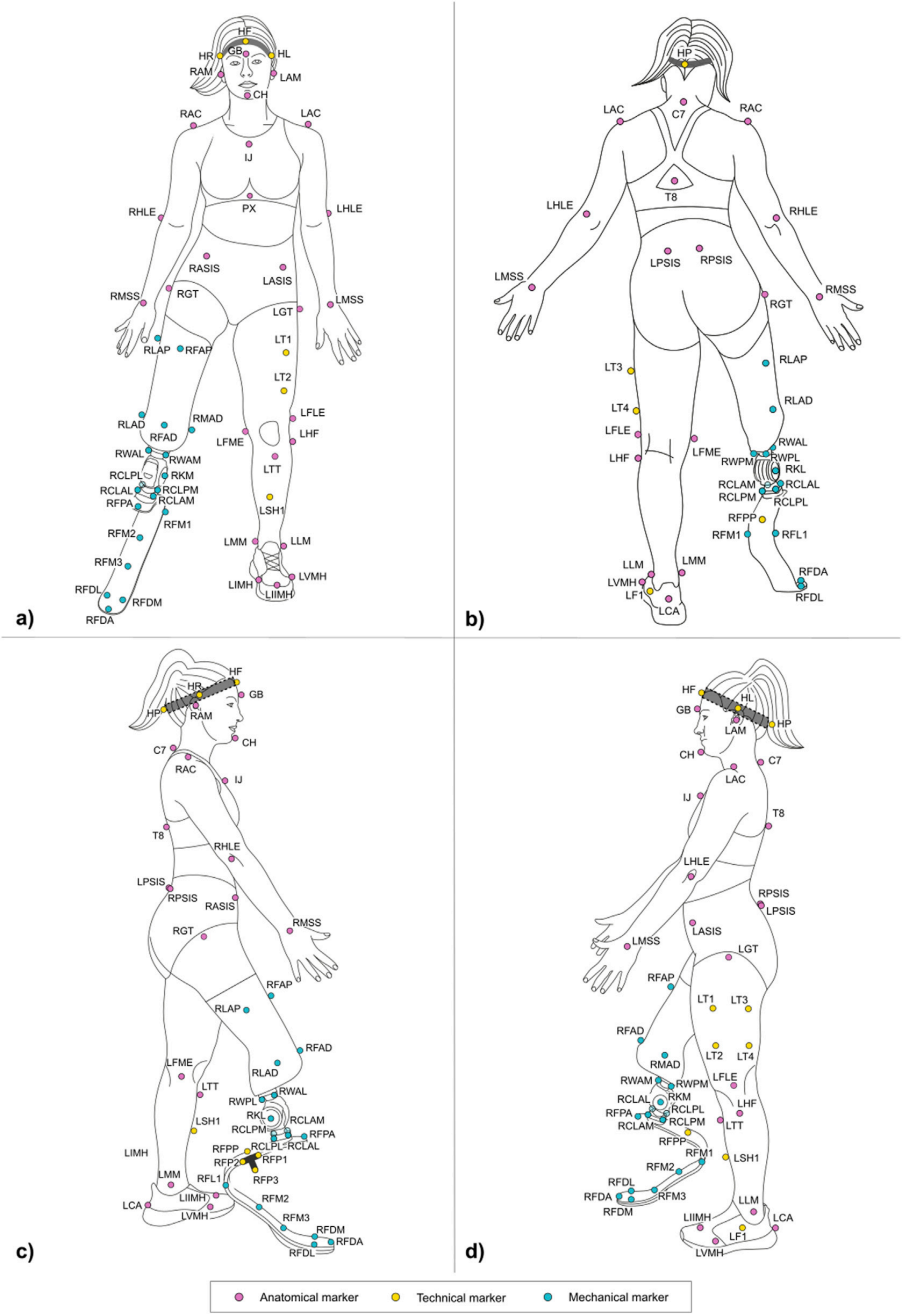
Frontal **(a)** posterior **(b)** lateral right **(c)** and lateral left **(d)** view of the full marker-set applied on an athlete with a TF amputation on the lower-limb. Markers can be anatomical (light magenta), technical (yellow), and mechanical (light blue). Head markers (HF, HR, HL, HP) are placed on an elastic headband.

**FIGURE 6:**
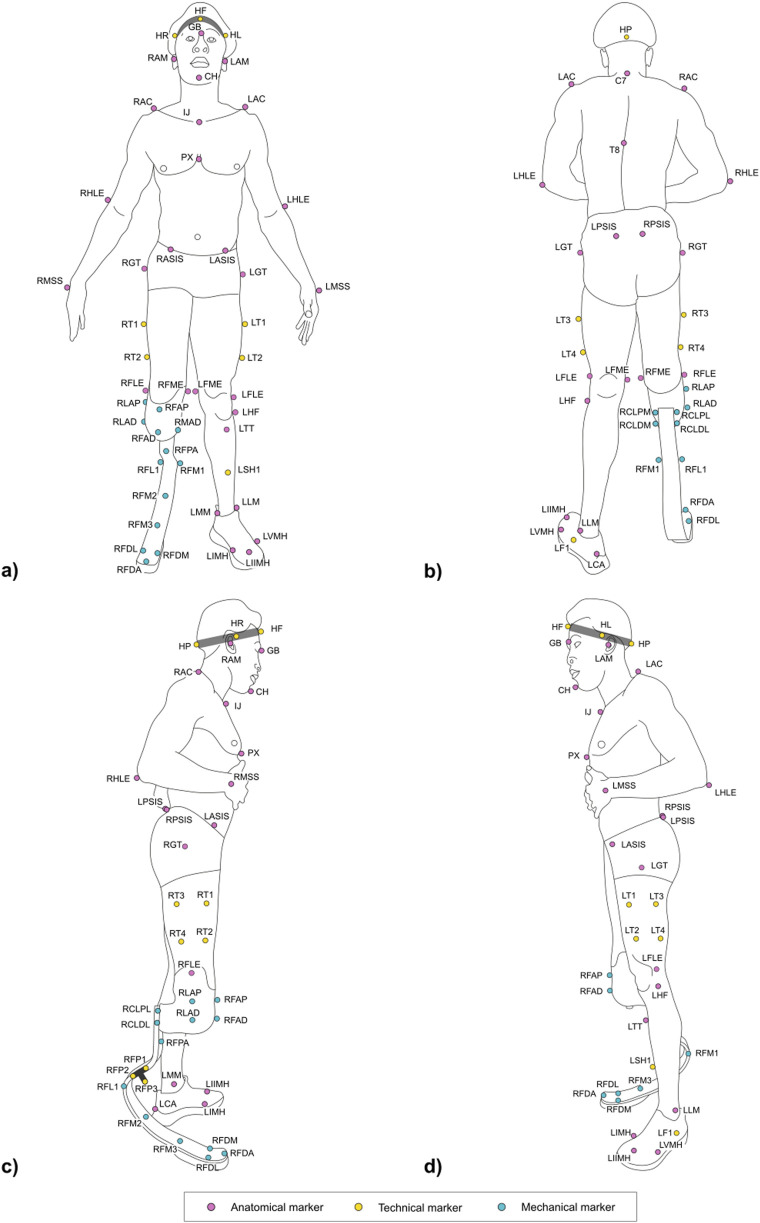
Frontal **(a)** posterior **(b)** lateral right **(c)** and lateral left **(d)** view of the full marker-set applied on an athlete with a TT amputation on the lower-limb. Markers can be anatomical (light magenta), technical (yellow), and mechanical (light blue). Head markers (HF, HR, HL, HP) are placed on an elastic headband.

Figures 5, 6 and Tables at the beginning of each 2.2.x paragraph and Supplementary Table 17 report the full marker-set for two athletes, one with a TF amputation at the right lower limb and one with a TT amputation at the right lower-limb. Markers can be either:

• *Physical*: when associated with a visible marker and attached to a landmark;
• *Virtual:* a body internal point, which can be reconstructed based on regression methods–e.g. the hip joint centre (Bell et al., 1990) – or arithmetical calculations–e.g. the knee joint centre, obtained as the midpoint between lateral and medial markers. Since those are internal points, they are not associated with any physically attached markers.

Markers can be further classified into three types, namely:

• *Anatomical:* physical or virtual marker associated with an anatomical landmark;
• *Mechanical:* physical or virtual marker associated with a geometrical landmark (also referred to herein as “mechanical landmark”) of a mechanical component;
• *Technical:* a physical marker needed to reconstruct the position of calibrated anatomical or geometrical landmarks through a static trial (Cappozzo et al., 2005); these markers are attached to the segment to ensure their traceability, i.e. they are not necessarily attached to a clearly defined landmark. A group of technical markers attached to the same segment is named as “technical cluster.”

Anatomical and mechanical markers can then pertain to three classes:

• *permanent*: a physical marker that will stay on the subject’s body or RSP throughout the whole data acquisition process. A marker pertains to this class if not otherwise specified.
• *Static*: a physical marker that is detached from the subject or the RSP after completion of a static (standing) calibration trial following the approach reported in (Cappello et al., 1997). These are denoted with an “S” in the Tables containing the marker list.
• *Wand-calibrated*: wand-calibrated markers are not physically placed on the subjects but are pointed with a marker-equipped wand of known geometry, following the approach reported in (Cappello et al., 1997). Landmarks palpated with the wand tip are then reconstructed with respect to the markers on the wand itself and, eventually, made solid with the segment cluster the pointed markers refer to. These are denoted with a “W” in the Tables containing the marker list.

**FIGURE 7:**
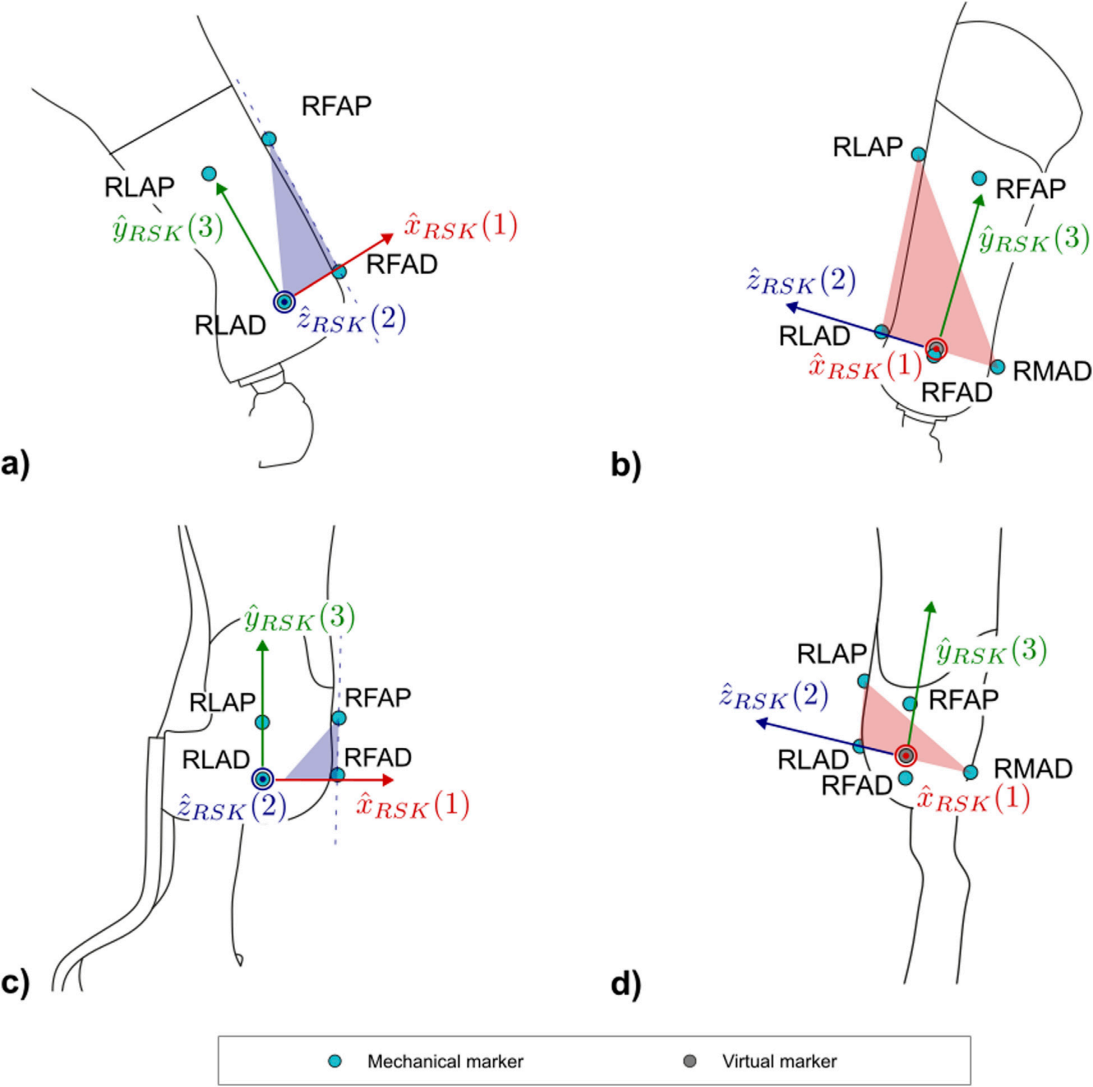
Markers and local coordinate system definitions of: **(a)** right lateral and **(b)** frontal views of the socket for PTFAs; **(c)** right lateral and **(d)** frontal views of the socket for PTTAs. Light blue markers are mechanical, while virtual calculated points are in grey.

**FIGURE 8:**
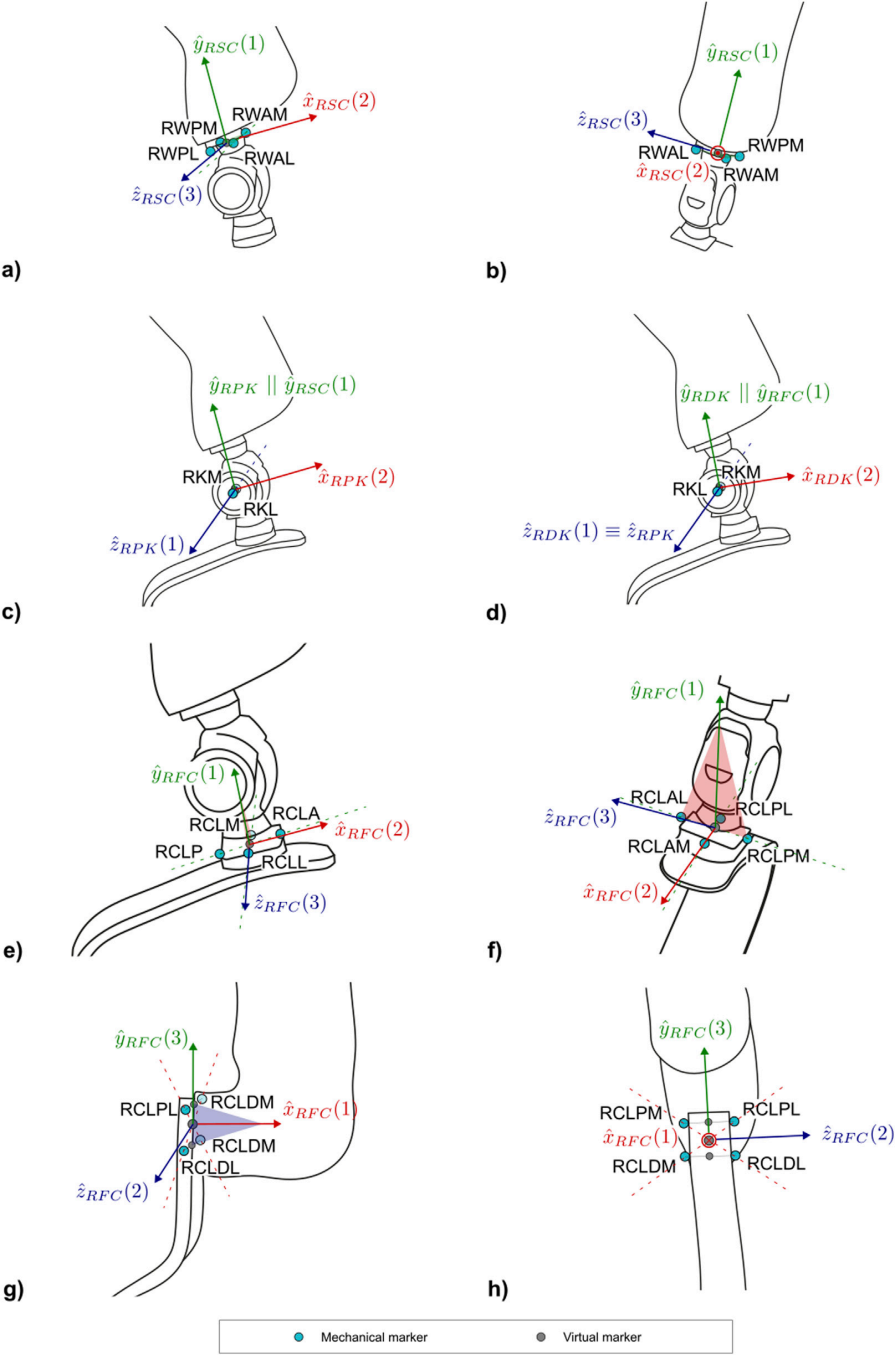
Markers and local coordinate system definitions of: **(a)** right lateral and **(b)** frontal views of the socket-clamp for PTFAs; **(c)** right lateral view of the proximal prosthetic knee and **(d)** distal prosthetic knee for PTFAs; **(e)** right lateral and **(f)** frontal views of foot-clamp for the PTFAs; **(g)** right lateral and **(h)** frontal views of the foot-clamp for PTTAs. Light blue markers are mechanical, while virtual calculated points are in grey.

**FIGURE 9:**
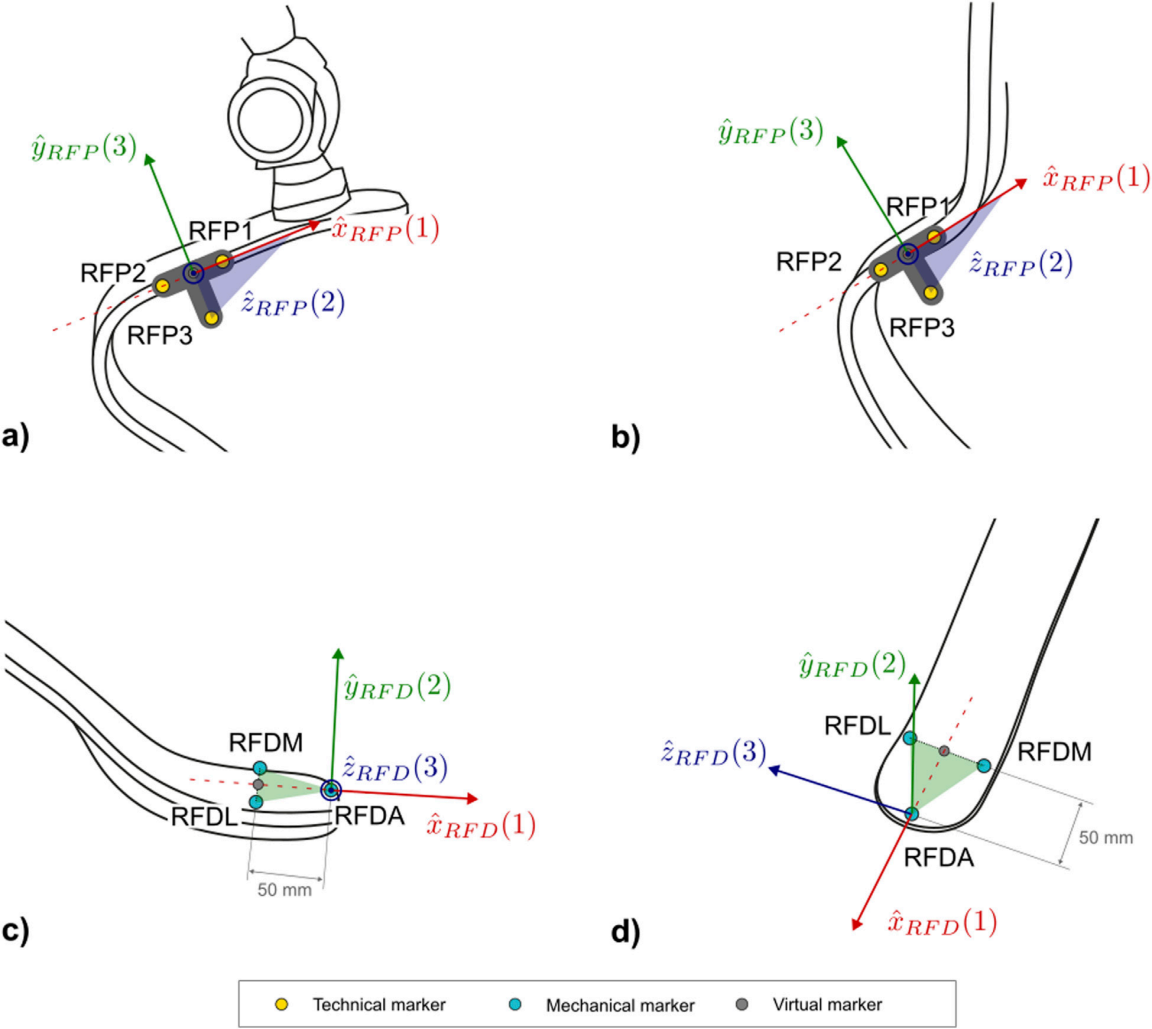
Markers and local coordinate system definitions of: the technical proximal running prosthetic foot (right lateral view) for TF **(a,b)** PTTAs; and **(c)** right lateral and **(d)** frontal views of the distal portion of the running prosthetic foot for the TF and PTTAs. Light blue markers are mechanical, while virtual calculated points are in grey.

**FIGURE 10:**
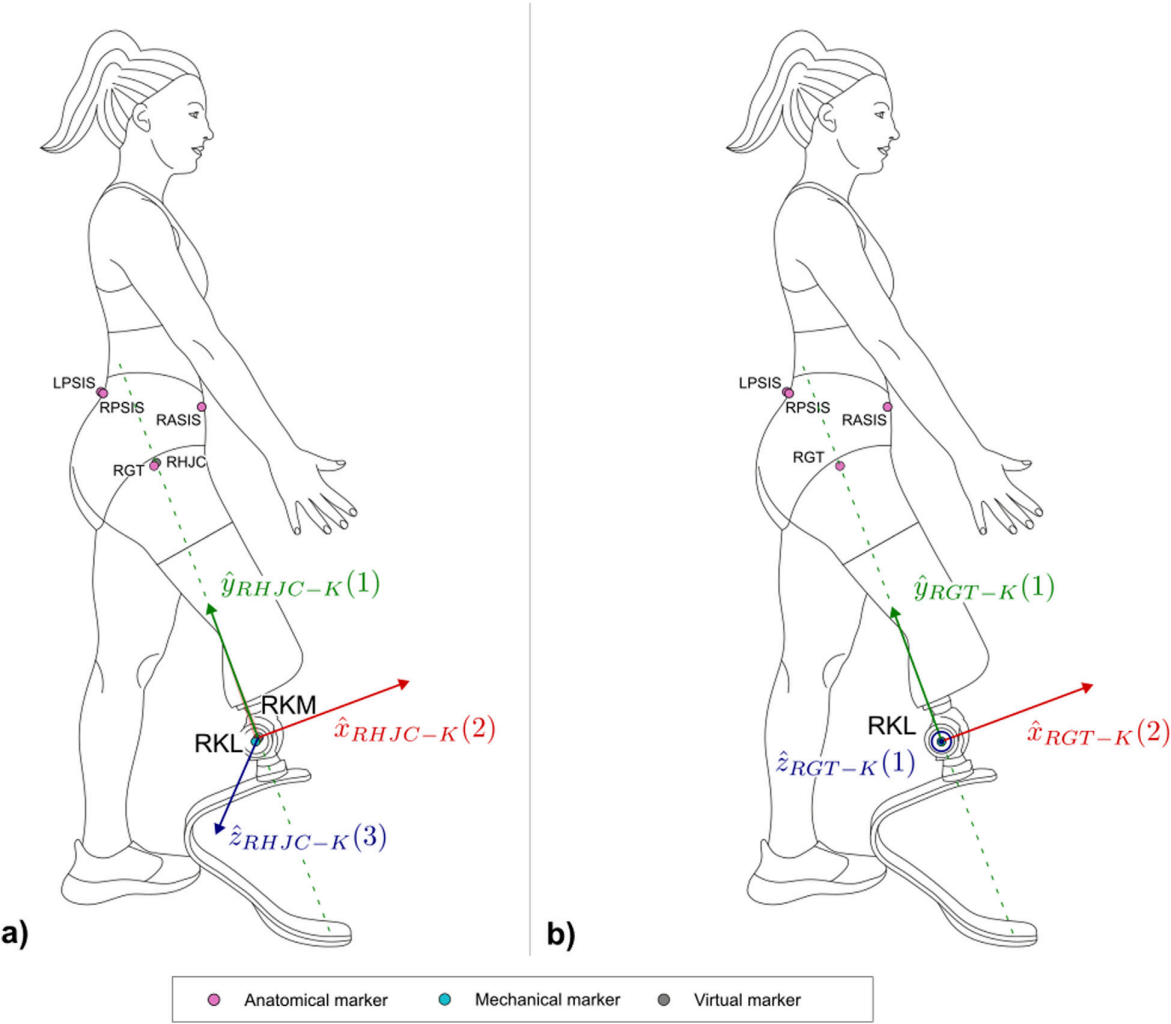
Right lateral view of the three-dimensional **(a)** and two-dimensional **(b)** systems built on the limb wearing the running specific prosthesis with the markers. Light magenta markers are anatomical, light blue markers are mechanical, while virtual calculated points are in grey.

The protocol follows the CAST approach (Cappozzo et al, 1995). Each subsection describing a segment and Supplementary Section 2 report the CSs definitions. To ease their understanding, Figures 7–10 provide a visual representation of CS construction. Axes x, y and z are coloured following the RGB code. In addition, the order of construction of the CS axes is indicated by a number in brackets next to the axis name: e.g. x (1), z (2), y (3) indicates that the first axis of the CS to be defined is x, followed by the definition of z, to conclude with the definition of y. Finally, relevant construction planes based on markers are also provided, with their colour matching that of the axis to which they are perpendicular.

#### 2.2.1 Socket

Lower limb sockets included in RSP have a bespoken shape to accommodate the specific athlete’s anatomy and component alignment and consist of a carbon fibre laminated frame that provides the mechanical strength to support the high demanding activities (Figure 4). The laminated frames can either fully enclose the residual limb in a closed structure or feature large anterior and/or posterior openings (“windows”) that expose an inner flexible socket made of thermoplastic material (e.g. ethylene-vinyl acetate).

In order to adapt to both closed and windowed sockets and account for the socket clamp placed behind it in case of PTTA, the reference system will be mainly constructed from markers placed on the lateral and medial sides of the socket, which normally have a cylindrical or conical shape (Table 1).

**TABLE 1:** List of markers used for the socket segment.

Marker	Marker type	Notes	Description
<s>FAP	Technical		Proximally (1/4 of the socket length) on the socket frontal axis
<s>FAD	Technical		Distally (3/4 of the socket length) on the socket frontal axis
<s>LAP	Technical		Proximally (1/4 of the socket length) on the socket lateral axis
<s>LAD	Technical		Distally (3/4 of the socket length) on the socket lateral axis
<s>MAD	Technical	S	Distally (3/4 of the socket length) on the socket medial axis
<s>GT	Anatomical	TF only	Greater trochanter (as defined for the sound limb)

Markers position depends on the knowledge of the socket frontal and lateral axes, as established by a certified prosthetist when the athlete dons the RSP. No established international standard exists that defines the frontal and lateral axes of the socket. To tackle this relevant limitation, a procedure is reported in Supplementary Section 3, based on current clinical practice and extensive discussions within the ISO Standardisation committees TC168 WG1 and WG3.

The definition of the socket CS (abbreviated as *SK* and shown in Figure 7) is based on the points LAP, LAD, MAD. The proposed definition of the CS ensures consistency with the 2D definition of the same CS: i.e., the longitudinal axis of the socket leans on the LAP-LAD-MAD defined plane and is perpendicular to x^SKG (Table 2).

**TABLE 2:** Socket coordinate system definition.

Axis/point	Formula	Description
Tag: <s> *SK*		
OSKG	$\frac{LAD+MAD}{2}$	Midpoint between LAD and MAD
x^SKG	$\frac{\left(LAP-LAD\right)\times \left(LAD-MAD\right)}{\left\|\left(LAP-LAD\right)\times \left(LAD-MAD\right)\right\|}$	Perpendicular to the quasi-frontal plane defined by LAP, LAD, and MAD, pointing forward (to be adapted for center and right sides – the formula is for right side)
z^SKG	x^SKG×FAP−FADx^SKG×FAP−FAD	Perpendicular to the quasi-sagittal plane defined by x^SKG and the line joining FAD to FAP
y^SKG	z^SKG×x^SKG	Perpendicular to the *zx* plane

#### 2.2.2 Socket-clamp–TF

Supplementary Figure 2 shows an example of socket clamp used in TF sockets. It is a squared, 4-screw adapter placed distally to the socket. This CS is essential for transferring ground reaction forces and moments to the interface between the clamp and the socket, to support bench tests definition.

The following markers are intended to be solid with the cluster placed on the socket composed by FAP, FAD, LAP, LAD, and MAD markers. Indeed WAM, WAL, WPM and WPL landmarks are wand-calibrated with respect to the socket technical cluster (Table 3).

**TABLE 3:** List of markers used for the socket-clamp segment in PTFAs.

Marker	Marker type	Notes	Description
<s>WAM	Mechanical	W	Screw of the socket connector – anterior-medial
<s>WAL	Mechanical	W	Screw of the socket connector – anterior-lateral
<s>WPM	Mechanical	W	Screw of the socket connector – posterior-medial
<s>WPL	Mechanical	W	Screw of the socket connector – posterior-lateral

The definition of the socket-clamp coordinate system (abbreviated as *SC* and shown in Figure 8) is based on the points WAL, WAM, WPL, WPM (Table 4).

**TABLE 4:** Socket-clamp coordinate system definition in PTFAs.

Axis/point	Formula	Description
Tag: <s> *SC*		
OSCG	$\frac{WAL+WAM+WPL+WPM}{4}$	Centroid of the points WAL, WAM, WPL and WPM
y^SCG	$\frac{\left(WAL-WPM\right)\times \left(WAM-WPM\right)}{\left\|\left(WAL-WPM\right)\times \left(WAM-WPM\right)\right\|}$	Perpendicular to the plane defined by WAL, WAM and WPM (to be adapted for center and right sides – this formula is for right side)
x^SCG	y^SCG×WAL−WAMy^SCG×WAL−WAM	Perpendicular to the plane defined by y^SCG and the line joining WAM to WAL, pointing forward
z^SCG	x^SCG×y^SCG	Perpendicular to the *xy* plane

#### 2.2.3 Prosthetic mechanical knee–PTFAs

Two coordinate systems have been defined to capture the flexion angle of the prosthetic mechanical knee: a proximal and a distal functional knee coordinate system (Kontaxis et al., 2009). Particular attention is given to defining the shared axis of flexion, which both coordinate systems have in common. This shared axis is the reason why the coordinate systems are referred to as “functional”. Notably, the markers used to define this axis (KL and KM–Table 5) are measured directly by the optoelectronic system and should not be reconstructed with techniques such as singular value decomposition relative to other segments (Cappozzo et al., 2005). This allows avoiding cluster deformation effects, with consequent marker trajectory mis-reconstruction, which may introduce undesired crosstalk in kinematics estimation.

**TABLE 5:** List of markers used for the prosthetic mechanical knee in PTFAs.

Marker	Marker type	Description
<s>KL	Mechanical	Placed laterally on the prosthetic knee centre of rotation
<s>KM	Mechanical	Placed medially on the prosthetic knee centre of rotation

##### 2.2.3.1 Proximal prosthetic knee functional CS

The Proximal Prosthetic Knee Functional CS is defined starting from the knee flexion axis in combination with the socket-clamp coordinate system (Table 4). The definition of the proximal functional knee coordinate system (abbreviated as *PK* and shown in Figure 8c) is based on the points KM, KL and the pseudo-vertical axis of the socket-clamp coordinate system (Table 6).

**TABLE 6:** Proximal prosthetic functional knee coordinate System definition.

Axis/point	Formula	Description
Tag: <s> *PK*		
OPKG	$\frac{KL+KM}{2}$	Midpoint between KL and KM
z^PKG	$\frac{KL-KM}{\left\|KL-KM\right\|}$	On the line joining KM to KL, pointing rightward (to be adapted for left and right sides – the formula is for right knee)
x^PKG	y^SCG×z^PKG	Perpendicular to the plane defined by *y* of the socket-clamp and the line joining KM to KL (pointing forward)
y^PKG	z^PKG×x^PKG	Perpendicular to the *zx* plane

##### 2.2.3.2 Distal prosthetic knee functional CS

The Distal Prosthetic Knee Functional CS is defined starting from the knee flexion axis and the foot-clamp CS (Table 12). The definition of the proximal functional knee coordinate system (abbreviated as *DK* and shown in Figure 8d) is based on the points KM, KL and the pseudo-vertical axis of the foot-clamp coordinate system.

Since the pylon and/or pyramid adapters proximal and distal to the prosthetic knee are not necessarily aligned, the flexion angle does not necessarily equal 0 when the mechanical knee is completely extended. The offset should be reported over the dynamic time history of the prosthetic knee flexion/extension angle (Table 7).

**TABLE 7:** Distal prosthetic functional knee coordinate system definition.

Axis/point	Formula	Description
Tag: <s> *DK*		
ODKG	$\frac{KL+KM}{2}$	Midpoint between KL and KM
z^DKG	$\frac{KL-KM}{\left\|KL-KM\right\|}$	On the line joining KM to KL, pointing rightward (to be adapted for left and right sides – the formula is for right knee)
x^DKG	y^FCG×z^DKG	Perpendicular to the plane defined by the *y* axis of the foot-clamp and the line joining KM to KL (pointing forward)
y^DKG	z^DKG×x^DKG	Perpendicular to the *zx* plane

#### 2.2.4 Running prosthetic foot clamp

##### 2.2.4.1 C-shaped RPF

C-shaped RPF can differ on clamp geometry depending on the manufacturer (Supplementary Figure 3). This protocol proposes strategies to adapt the biomechanical model to as many clamp types as possible, so to identify the axis normal to the clamp plane and the tangent to the proximal portion of the RPF lying on the sagittal symmetry plane of the RPF.

In the case of a sliding pyramid adapter foot-clamp (Supplementary Figure 3a) such as the one of Ottobock Runner RPF, markers are placed on a custom 3D printed “crossed-shaped collar support” (Supplementary Figure 3b), which is rigidly connected to the clamp with set screws. Once in place, the markers on the collar lay on a plane parallel to the clamp-RPF interface. The collar support is needed as there would not be enough room to directly attach markers on the foot-clamp support. The STL file to replicate the collar support for the Ottobock Runner RPF is provided as an example in Supplementary Section 1.1.

In case of 4-hole pyramid adapter foot-clamps (Supplementary Figure 3b-d), mechanical landmarks needed to build the local CS are wand-calibrated following the configuration given in Table 4, as the interface between the foot-clamp and the foot is secured with screws via through-holes. These landmarks are calibrated relative to a custom 3D printed T-shaped rigid support that host three technical markers and that is attached laterally to the RPF immediately posterior to the foot clamp. A technical Coordinate System can be defined for this T-shaped support, named Technical Proximal Running Prosthetic Foot, as reported in Section 2.2.6.1. The STL file to replicate the T-shaped support is provided in Supplementary Section 1.1.

Markers <s>CLA, <s > CLM, <s> CLL and <s> CLP are then obtained as follows (the <s> is omitted in the formulas for simplicity) (Table 8):

$$CLA=\frac{CLAL+CLAM}{2}\;CLM=\frac{CLAM+CLPM}{2}$$

$$CLL=\frac{CLAL+CLPL}{2}\;CLP=\frac{CLPL+CLPM}{2}$$

**TABLE 8:** List of markers for the foot-clamp segment in PTFAs.

Marker	Marker type	Notes	Description
<s>CLL	Mechanical		Foot-Clamp: lateral
<s>CLA	Mechanical		Foot-Clamp: anterior
<s>CLM	Mechanical		Foot-Clamp: medial
<s>CLP	Mechanical		Foot-Clamp: posterior
<s>CLAL	Mechanical	W	Anterior lateral screw of the foot-clamp plaque
<s>CLPL	Mechanical	W	Posterior lateral screw of the foot-clamp plaque
<s>CLAM	Mechanical	W	Anterior medial screw of the foot-clamp plaque
<s>CLPM	Mechanical	W	Posterior medial screw of the foot-clamp plaque

The definition of the foot-clamp coordinate system for PTFAs (abbreviated as *FC* and shown in Figures 8e,f) is based on the points CLA, CLM, CLL, CLP (Table 9).

**TABLE 9:** Foot-clamp coordinate system definition in PTFAs.

Axis/point	Formula	Description
Tag: *<s> FC*		
OFCG	$\frac{CLA+CLL+CLM+CLP}{4}$	Centre of mass of the foot-clamp markers
y^FCG	$\frac{\left(CLL-CLM\right)\times \left(CLA-CLP\right)}{\left\|\left(CLL-CLM\right)\times \left(CLA-CLP\right)\right\|}$	Perpendicular to the plane defined by the line joining CLM to CLL, and the line joining CLP to CLA, pointing upward (to be adapted for center and right sides – this formula is for right prosthetic foot)
x^FCG	y^FCG×CLL−CLMy^FCG×CLL−CLM	Perpendicular to the plane defined by y^FCG and the line joining CLM to CLL
z^FCG	x^FCG×y^FCG	Perpendicular to the *xy* plane

##### 2.2.4.2 J-shaped RPF

For J-shaped RPF, 4 markers are used to define the foot-clamp CS. Markers are applied to the lateral and medial side of the RPF in its most proximal part of the RPF (Figures 4, 6) at the same height of the two screws that connect the foot to the socket posterior box (Table 10).

**TABLE 10:** List of markers used for the foot-clamp segment in PTTAs.

Marker	Marker type	Notes	Description
<s>CLPL	Mechanical		Foot-Clamp: lateral proximally
<s>CLDL	Mechanical		Foot-Clamp: lateral distally
<s>CLPM	Mechanical		Foot-Clamp: medial proximally
<s>CLDM	Mechanical		Foot-Clamp: medial distally
<s>WP	Mechanical	W	Screw to connect the prosthetic foot to the socket – proximal screw
<s>WD	Mechanical	W	Screw to connect the prosthetic foot to the socket – distal screw

The definition of the foot-clamp coordinate system (abbreviated as *FC* and shown in Figures 8g,h) is based on the points CLPM, CLPL, CLDM, CLDL (Table 11).

**TABLE 11:** Foot-clamp coordinate system definition in PTTAs.

Axis/point	Formula	Description
Tag: <s> *FC*		
OFCG	$\frac{CLPM+CLPL+CLDM+CLDL}{4}$	Centroid of the clamp markers
x^FCG	$\frac{\left(CLPM-CLDL\right)\times \left(CLPL-CLDM\right)}{\left\|\left(CLPM-CLDL\right)\times \left(CLPL-CLDM\right)\right\|}$	Perpendicular to the plane defined by CLPL, CLPM and CLDL and CLDM, pointing forward (to be adapted for left and right sides – this formula is for right prosthetic foot)
P1G	$\frac{CLPL+CLPM}{2}$	Midpoint between CLPL and CLPM
P2G	$\frac{CLDL+CLDM}{2}$	Midpoint between CLDL and CLDM
z^FCG	x^FCG×P1−P2x^FCG×P1−P2	Perpendicular to the plane defined by x^FCG and the line joining P_2_ to P_1_, pointing rightward
y^FCG	z^FCG×x^FCG	Perpendicular to the *zx* plane

#### 2.2.5 Distal running prosthetic foot

The definition of the distal portion of the running prosthetic foot coordinate system (abbreviated as *FD* and shown in Figure 9b) is based on the points FDA, FDM, FDL (Tables 12, 13).

**TABLE 12:** List of markers used for the distal running prosthetic foot.

Marker	Marker type	Description
<s>FDA	Technical	The most distal point (tip) of the prosthetic foot
<s>FDM	Technical	Placed medially on the foot tip, 50 mm proximally from FDA
<s>FDL	Technical	Placed laterally on the foot tip, 50 mm proximally from FDA

**TABLE 13:** Distal prosthetic foot coordinate system definition.

Axis/point	Formula	Description
Tag: <s> *FD*		
OFDG	FDA	
$O_{1}$	$\frac{FDM+FDL}{2}$	Midpoint between FDM and FDL
x^FDG	$\frac{\left(FDA-O_{1}\right)}{\left\|\left(FDA-O_{1}\right)\right\|}$	On the line joining O_1_ and FDA, pointing forward
y^FDG	xFDG×FDM−FDLxFDG×FDM−FDL	Perpendicular to the plane defined by FDA, FDM and FDL (to be adapted for left and right sides – this formula is for right prosthetic foot)
z^FDG	x^FDG×y^FDG	Perpendicular to the *xy* plane

#### 2.2.6 Auxiliary CS

##### 2.2.6.1 Technical proximal running prosthetic foot

As anticipated in Section 2.2.4.1, this technical coordinate system is built on the technical cluster consisting of the markers FP1, FP2 and FP3 (Table 14). This CS (Figures 9a,b) is not used to compute any joint kinematics during the exercise, but shall the prosthetist change the position of the foot-clamp with respect to the RPF, by keeping the triad fixed on the RPF across the trials, this system allows measuring differences among alignments due to different configurations. Additionally, in case of visibility loss for other markers on the foot-clamp or wand-calibrated markers of the foot-clamp, this cluster triad enables the reconstruction of those markers’ trajectories.

**TABLE 14:** List of markers used for the technical proximal portion of running prosthetic foot segment.

Marker	Marker type	Notes	Description
<s>FPA	Technical	TF only S	Foot proximal anterior - Placed as anteriorly as possible on the mid-line of the prosthetic foot
<s>FPA	Technical	TT only	Foot proximal anterior – Placed on the mid-line of the prosthetic foot two fingers below the clamp
<s>FP1	Technical		Placed on a T-frame clamped to the prosthetic foot, proximally to clamp
<s>FP2	Technical		Placed on a T-frame clamped to the prosthetic foot, distally to clamp on the same line of FP1
<s>FP3	Technical		Placed on a T-frame clamped to the prosthetic foot, perpendicularly downwards to the line joining FP1 and FP2

The definition of the technical system of the proximal running prosthetic foot coordinate system (abbreviated as *FP* and shown in Figures 9a,b) is based on the points FP1, FP2, FP3 (Table 15).

**TABLE 15:** Technical proximal RPF coordinate system definition.

Axis/point	Formula	Description
Tag: <s> *FP*		
OFPG	$\frac{FP1+FP2}{2}$	Midpoint between FP1 and FP2
x^FPG	$\frac{FP1-FP2}{\left\|FP1-FP2\right\|}$	Line joining FP2 to FP1, pointing forward
z^FPG	xFPG×OTFG−FP3xFPG×OTFG−FP3	Perpendicular to the plane defined by FP1, FP2 and FP3 (to be adapted for left and right sides – this formula is for right prosthetic foot)
y^FPG	z^FPG×x^FPG	Perpendicular to the *zx* plane

The <s>FPA markers are not intended to be used for CS definition but are considered as part of the technical cluster of the RPF in case of marker’s trajectory occlusion. Moreover, the relative motion of the <s>FDA marker (on the tip of the distal portion of the RPF) with respect to the <s>FPA can be informative of the actual RPF compression.

##### 2.2.6.2 Equivalent leg systems

The two following CSs are defined to build an equivalent mechanical segment of the thigh in PTFAs as a whole and to attempt modelling an equivalent mechanical axis used for alignment of the RSP components during production and bench tests by the prosthetist, especially for socket testing (Migliore et al., 2021). Moreover, tracking the trajectory of the RPF tip with respect to the CSs defined in the following paragraphs would provide a measure of the “feeling” of the athletes during their performances (Hansen et al., 2004). The first CS is defined as a 3D system, whereas the second is a 2D system to be used to compare data gathered via stereophotogrammetry with those collected with high rate and high-resolution video recordings.

###### 2.2.6.2.1 HJC-K coordinate system definition (3D system)

The definition of the HJC-K coordinate system (Figure 10a) is based on the points HJC, KM, KL (Table 16).

**TABLE 16:** Auxiliary 3D coordinate system definition based on the line connecting the hip joint centre to the prosthetic knee centre.

Axis/point	Formula	Description
Tag: *<s> HJC-K*		
OHJC−KG	$\frac{KM+KL}{2}$	Midpoint between KM and KL
y^HJC−KG	HJC−OHJC−KGHJC−OHJC−KG	On the line joining the prosthetic knee centre to the HJC
x^HJC−KG	y^HJC−KG×KL−KMy^HJC−KG×KL−KM	Perpendicular to the plane defined by HJC, and the line joining KM to KL, pointing forward (to be adapted for left and right sides – the formula is for right knee)
z^HJC−KG	x^HJC−KG×y^HJC−KG	Perpendicular to the *xy* plane

###### 2.2.6.2.2 GT-KL coordinate system definition (2D system)

This reference system, analogous to the previous one, is useful for a direct comparison with a video analysis, where the traces of HJC and the midpoint between KM and KL are not available, but an approximation of their external projections are rather captured (i.e., GT and KL).

The definition of the GT-KL coordinate system (Figure 10b) is based on the points GT, KL (Table 17).

**TABLE 17:** Auxiliary 2D coordinate system definition based on the line connecting the lateral approximation of the hip joint centre (i.e., the great trochanter) to the lateral projection of the prosthetic knee centre (i.e., the lateral knee marker).

Axis/point	Formula	Description
Tag: <s> *GT-KL*		
OGT−KLG	KL	Coincident with KL
y^tempG	GT−OGT−KLGGT−OGT−KLG	On the line joining KL to GT
y^GT−KLG	y^temp,xG y^temp,yG 0y^temp,xG y^temp,yG 0	Projection of the temporary y axis on the Laboratory sagittal plane, defined by the running direction (*x*_*G*_) and gravity (-*y*_*G*_)
z^GT−KLG	$\left[\begin{array}{ccc} 0 & 0 & 1 \end{array}\right]$	Parallel to the Laboratory CS lateral axis, i.e. normal to the running direction and gravity
x^GT−KLG	y^GT−KLG×z^GT−KLGy^GT−KLG×z^GT−KLG	Perpendicular to the *yz* plane

### 2.3 Kinematics–cardan sequences

Joint kinematics is obtained as decomposition of the relative rotations between adjacent segments and following the International Society of Biomechanics recommendations (Wu et al., 2005; 2002; Wu and Cavanagh, 1995), based on the Cardan convention originally proposed by Grood and Suntay for the lower limb joints to associate a clinical meaning to the obtained rotations (Grood and Suntay, 1983). Detailed definition of the Cardan sequences to decompose the relative rotation matrices are given in Supplementary Table 18. The *zx’y’’* sequence was used to decompose all joint relative rotation matrices, except for ankle and *virtual ankle* (i.e., the relative orientation of the distal portion of the RPF with respect to the foot-clamp coordinate system) where the chosen sequence was *zy’x’’*.

Segment orientation is obtained decomposing the rotation matrix of each local embedded coordinate system with respect to the ground coordinate system and considering a roll-pitch-yaw convention.

### 2.4 Data collection procedure

The preparation process starts by drawing the main socket lines as described in paragraph 2.2.1 and the subsequent marker placement on the RSP (socket, knee -if any- and RPF). Afterward, the athlete’s skin is prepared to ensure proper adhesion of tapes and markers by gently scrabbing with alcoholic wipes. Pre-taping and Kinesiotape are then applied to the athlete’s skin and the areas of the running shoes over anatomical and technical landmarks. Following this step, anatomical and technical landmarks are palpated and marked with a pen and subsequently markers are physically placed on the athlete, using a highly adhesive double-sided tape. Palpation takes advantage of the athlete’s verbal feedback when dealing with anatomical landmarks of the foot in the shoe. The athlete then undergoes a warm-up session, which is crucial for becoming accustomed to the markers and allowing full focus on performance. Experience suggests that placing retroreflective markers before the warming up reduced their detachment, rather than letting them warm up and placing markers subsequently because of perspiration. After the warm-up, markers are checked and adjusted, if necessary. The total preparation time, excluding the warm-up, is approximately 25 min. However, since the prosthesis preparation can occur independently of the athlete’s direct involvement, the impact on the athlete’s preparation time is around 20 min.

Static measurements are then collected with the athlete standing upright. After the static standing trial, an operator proceeds by collecting separate static trials for each of the wand-calibrated markers, using a marker-equipped wand (5 min). Finally, dynamic assessments are performed, with their duration depending on the specific data to be captured. Appropriate resting time must be granted among trials based on the athlete’s and their coach feedback.

### 2.5 Data analysis

Marker trajectories collected during standing and running are pre-processed as per motion capture routinary pipeline: reconstructed, labelled and gap filled and smoothed within the software of the optoelectronic system, e.g. Vicon Nexus for our set-up (version 2.13+, Vicon Motion Systems Ltd., Oxford, UK), as well as the manual foot-strike and foot-off event detection.

Once these preliminary steps are completed, 1) virtual and calibrated markers are localized with respect to their relevant clusters using regression methods (Bell et al., 1990), and data from wand trials (Cappello et al., 1997), respectively. Then, 2) they are reconstructed in a static trial containing all the physical and calculated markers. Thereafter, 3) all virtual and static-only markers from the processed static trial are reconstructed in dynamic trials using the singular value decomposition approach (Cappozzo et al., 2005). Finally, 4) local coordinate systems construction and kinematics calculations are completed for each static and dynamic trial, following the definitions provided in this paper. Joint kinematics are time-normalized over the stride percentage (with 0% and 100% being subsequent foot-strike of the same side).

### 2.6 Olympia open-source software

Steps 1) to 4) as described in Section 2.5, are complex and would typically require the implementation of a custom-made software. This is a time-consuming operation and would require specific technical knowledge. This requirement can limit the diffusion of the protocol among the interested researchers. For this reason, alongside the manuscript, we are providing a MATLAB open-source implementation of the software, called Olympia, needed to complete the steps.

Assuming the data collection procedure described in Section 2.4, the Olympia software was designed to compute segmental and joint kinematics based on the definition of the biomechanical model, starting from pre-labelled static, calibration, and dynamic files. The system incorporates sufficient flexibility and adaptability to address the typical challenges encountered during real-life data collection.

Specifically, during the same data collection session, the RSP setup may change between trials for clinical or research purposes. This results in a change of configuration, requiring the entire data collection procedure to be restarted as described. Also, even if the prosthetic configuration remains unchanged, it is possible that one or more markers detach from the participant’s skin or from the RSP. In such cases, a new static trial must be collected. If a detached marker belongs to a cluster associated with wand-calibrated markers, those wand-calibrated markers must also be recalibrated after the new static trial.

### 2.7 Example dataset

To ensure that Researchers can practice with the Olympia software, an exemplary dataset on sprinting tests is provided alongside the source codes.

Specifically, the data collection protocol was used as part of the standard assessment routine and training during the 2023–2024 Paralympic season to support certified prosthetists in setting up the RSP for the elite sprinters in preparation of the Paralympic Games in Paris. The written consent to diffuse their data together with the Olympia software was obtained by two gold medallists, respectively with a TF and TT amputation.

The TF (female, mass: 55 kg, height: 1.60 m) is a T63 100 m medallist in Paris 2023 World Para Athletics Championships, who uses a 1E91 Standard Runner Cat 4 RPF (Ottobock, Germany) and 3S80 monoaxial prosthetic knee joint (Ottobock, Germany) on her left prosthetic leg. With reference to (Migliore et al., 2021), the socket tilt in the sagittal plane relative to the gravity was 15°.

The TT (male, mass: 85 kg, height: 1.87 m) is a T64 100 m medallist in Paris 2023 World Para Athletics Championships, who used as RPF a Sprinter Cat 4 (Ottobock, Germany) on his left prosthetic leg.

Tests were performed and recorded at the “Olympia SmartTrack” installed at the “Palaindoor Padova”, which consists of: (i) a 13.0 × 7.0 × 3.5 m^3^ portal to carry the (ii) 10-IR camera stereophotogrammetric system (Vicon Vantage V5, Vicon Motion Systems Ltd., Oxford, UK), and (iii) nine force plates (two BMS400600 and seven BMS600900, AMTI Advanced Mechanical Technology, Inc., Watertown, MA, USA) (Mistretta et al., 2022).

Both athletes were asked to run over a 60 m straight path. The camera configuration ensures a capture volume of approximately 7.0 × 3.0 × 2.0 m^3^. The portal and the force plates are positioned 30 m from the start line to allow for the collection of athletes’ steady-state running data.

## 3 Results

### 3.1 Olympia software architecture and procedure

**FIGURE 11:**
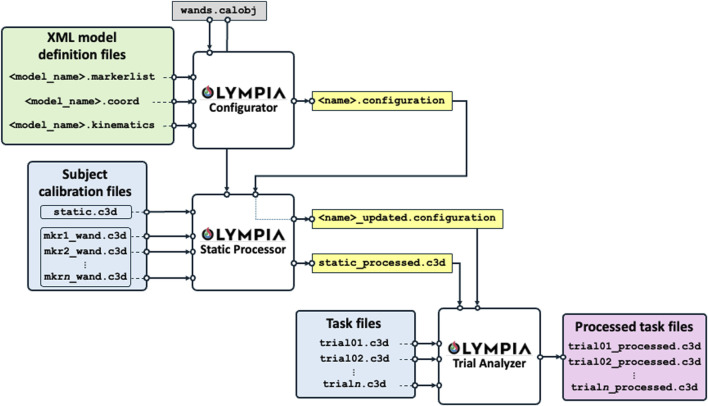
Software data and processing flow. The green box contains the proposed model definitions (or given according to any other biomechanical model to be adopted). The white boxes are the functional blocks of the software to process the data. The light blue boxes report the collected data to be analysed, the yellow boxes are the intermediate outputs, and the pink box reports the final processed task outputs.

The Olympia Software is publicly available at the URL https://www.doi.org/10.5281/zenodo.15537123. While referring the reader interested in the full details to Supplementary Section 7, we reported herein a brief explanation of the software architecture. Specifically, the processing flow is shown in Figure 11.

The software reads the “XML Model Definition Files” and the specific session C3D files. The XML Model Definition Files are provided preconfigured with the software, based on the protocol described in Section 2, embedding the information regarding the marker-set, the body segments, the coordinate system definition, and the kinematic chain (proximal and distal coordinate systems and Euler/Cardan sequences). XML files can be customised by expert users to adapt to their specific needs, if deemed necessary. C3D files are the de-facto standard used for motion analysis collection by most commercial marker-based systems.

The XML Model Definition Files and the C3D files are processed through three main functional modules: the configurator, the static processor, and the dynamic trial analyser.

The *Olympia Configurator* is a Graphical User Interface (GUI) that reads the XML Model Definition Files and the geometry of the calibration object and generates a “XML Configuration file” which adapts the general XML Model Definition Files to the specific subject under analysis, e.g. by considering the specific level of amputation and RPF model. Importantly, the “XML Configuration file” is the implementation of a key concept of the Olympia software, namely the “subject configuration”. Given a participant involved in a data collection activity (*session*), this is uniquely identified by the subject identification number and the date of the session. During a session, the subject may be asked to perform tests using different devices: e.g., to compare different prostheses or to run with alternative prosthetic alignments or RPF, each associated with a specific marker-set and reference systems pertaining to the protocol described in Section 2. Whenever the configuration of the subject under analysis changes (e.g., testing different alignments of a Running Specific Prosthesis, or testing different Running Prosthetic Feet), a new *configuration* is defined within the same session, i.e. a new “XML Configuration file” must be generated. For instance, the analysis of data gathered from the assessment of a subject using two prosthetic alignments A and B, possibly requiring a different marker positioning, will call for one XML file named “configuration A” and a second XML file named “configuration B″, respectively. However, when a new static acquisition is required within the same session, possibly due to one or more markers detached from the subject with their consequent repositioning, this does not correspond to a new configuration but rather to an “updated” registration of the same configuration. This is implemented in the Olympia Static Processor.

The *Static Processor* is a second GUI which accepts the configuration file, the C3D files from static trial and the wand calibrations, and the descriptor of the calibration object (i.e., the marker equipped wand). It generates a processed static trial embedding all physical, virtual, and calibrated reconstructed markers. Also, if one or more markers detach from the participant’s skin or from the RSP, the Static Processor allows the user to generate the “updated configuration file”.

Finally, the *Trial Analyzer* is a third GUI and final block of the software which fuses the information gathered from the processed static trial and configuration file to analyse the dynamic trials and return new C3D files enriched with the kinematics and all the virtual points reconstructed.

The system’s modularity allows users to easily adapt or extend models by editing XML files, making it broadly applicable across various motion analysis protocols.

### 3.2 Presentation of the exemplary dataset

The described procedures and software were used to process the example data, which are provided along with the software (available at https://www.doi.org/10.5281/zenodo.15537123). Data comprehend, per each participant: an unprocessed C3D file containing the data gathered from the static calibration trial, the C3D files containing the wand calibration data, the XML configuration file, the C3D files containing the data gathered from the dynamic trials, and the relevant processed C3D files.

**FIGURE 12:**
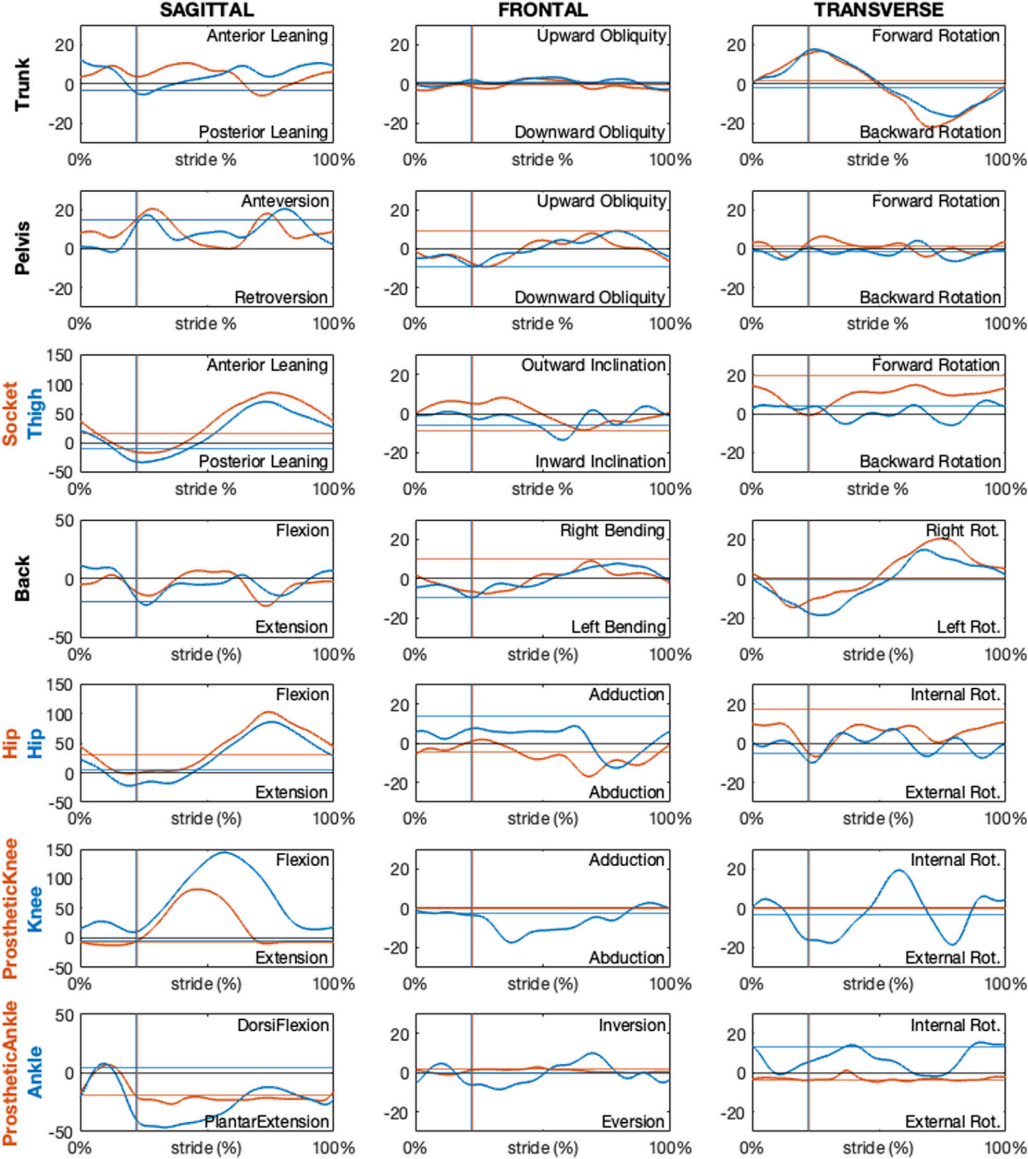
Sagittal, frontal and transverse absolute and relative kinematics obtained with the proposed model for a TF amputee during running. Kinematics, reported in blue for non-prosthetic (right) and in dark orange for prosthetic (left) sides, are time normalized over the stride percentage. Horizontal lines show the static kinematics. Vertical lines show the foot-off instants.

**FIGURE 13:**
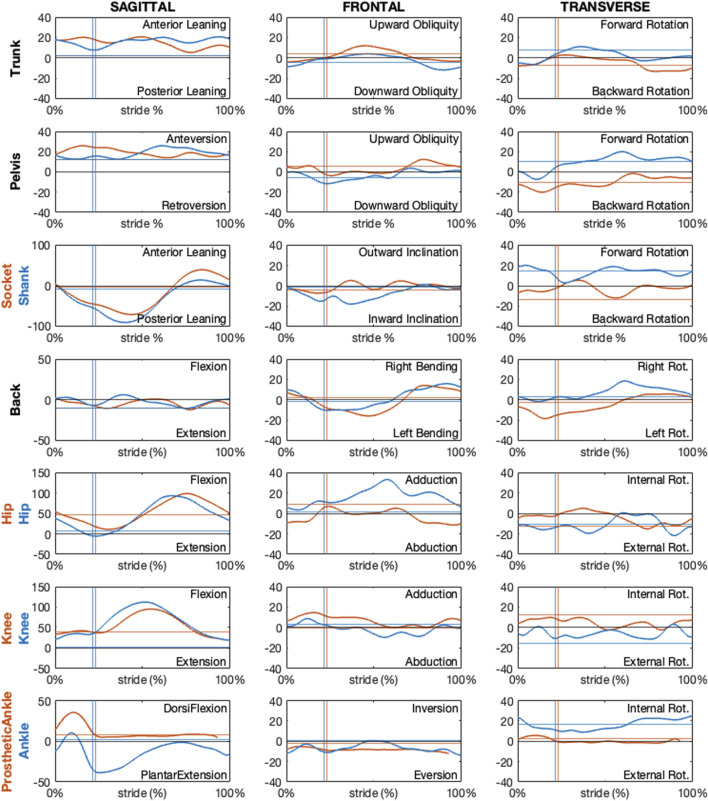
Sagittal, frontal and transverse absolute and relative kinematics obtained with the proposed model for a TT amputee during running. Kinematics, reported in blue for non-prosthetic (right) and in dark orange for prosthetic (left) sides, are time normalized over the stride percentage. Horizontal lines show the static kinematics. Vertical lines show the foot-off instants.

The following figures present paradigmatic examples of the kinematics obtained according to the proposed protocol for both the PTFA (Figure 12) and PTTA (Figure 13). Kinematics is given as decomposed on the sagittal, frontal, and transverse planes arranged in columns and different segment orientation and joint angles are arranged on rows, as recommended in (Benedetti et al., 2017) and normalized over the percentage of the stride. Foot-off events are highlighted with dashed vertical lines. To allow the reader appreciating the kinematics variations during the breaking and propulsion phases of sprinting, kinematics was also normalized over the percentage of the stance phase and presented on Supplementary Figure 9 and Supplementary Figure 10, respectively for the PTFA and PTTA. Positive rotation are as follows: anterior leaning and flexion (dorsal flexion in case of the ankle); adduction; internal rotation.

Regarding the PTFA, the running speed was 6.7 m/s. For the PTTA, the running speed was 8.7 m/s.

Kinematics obtained according to the proposed protocol correctly highlights a null prosthetic knee extension in stance and at terminal swing, as well as a null prosthetic knee abduction-adduction and internal-external rotation for the PTFA. Furthermore, the dorsal-plantar flexion of the prosthetic ankle is neutral (i.e. the same as the static asset) during late stance and swing phases, for both the PTFA and PTTA. It is worth noting that neutral dorsal-plantar flexion of the prosthetic ankle is not a null angle, even during the static trial (continuous horizontal lines in Figures 12, 13), since the distal portion of the RPF can be oriented such that its *x*-axis points slightly upwards or downwards with respect to the proximal segment used to define the joint. The extent to which the static plantar/dorsiflexion angles differ from zero depends on the considered RPF model. Abduction-adduction and internal-external rotation of the prosthetic ankle are neutral along the whole stride, with the deviations from the static asset possibly quantifying the vibration of the RPF.

## 4 Discussion

This study provides a standardized protocol for analysing the kinematics of running in PTFAs and PTTAs, while a full biomechanical analysis will be possible by integrating force plates and inertial parameters calculation for each segment. Unlike previous studies, the proposed definitions are not based on placing markers on landmarks identified on the prosthesis by their similarities with the sound limb (Kent and Franklyn-Miller, 2011). This allows the protocol to be applied to any RSP configuration, regardless of the relative position and orientation of the RSP components (i.e., sockets, adapters, pylons, prosthetic knees and prosthetic feet). The adoption of standardized local coordinate systems possibly facilitates the understanding of segmental and joint kinematics across different prosthesis configurations and studies (Sawers and Hahn, 2010), informing prosthetists and coaches in optimizing prosthesis setups for competition.

Defining one or more coordinate systems (CSs) for each prosthetic segment is crucial for both biomechanical analysis and bench/*in silico* testing. This paper addresses the lack of a standardised motion capture protocol for informing mechanical bench tests that can reproduce real-world conditions, and assess the functional characteristics of components specific to running (Gariboldi et al., 2025a; 2025b; 2022). Often, multiple CSs are needed for the same segment to meet different requirements. For example, in the case of transfemoral amputation, at least 3 CSs can be defined for the socket: (i) one based on the residual limb, which is considered as solid with the socket itself, (ii) one accounting for the affected hip and prosthetic knee joint, and (iii) one considering the position and orientation of the socket-clamp system. While the first is essential for defining the joint rotations of the affected hip, the second and third are necessary for correct socket alignment in a test bench environment, ensuring an accurate reproduction of *in-vivo* conditions in an *in-vitro* setting.

As previously mentioned, the definition of local reference systems follows the recommendations of the International Society of Biomechanics. The proposed CSs were developed after extensive discussions with a multi-professional group–including two prosthetists, three test engineers, and three biomechanists–and over 20 *in-vivo* testing sessions with multiple prosthetic configurations and socket mechanical bench testing (Gariboldi et al., 2025a; Gariboldi et al., 2025b; Gariboldi et al., 2022; Petrone et al., 2020). This approach ensures repeatability and enables a systematic investigation of the athlete-prosthesis interaction, while also promoting the integration of *in-vivo* biomechanics data into *in-vitro* (e.g., bench tests) and *in silico* (e.g., Finite Element Method) simulations. Such integration contributes to creating more realistic testing environments, ultimately leading to safer and more effective prosthetic designs.

One of the biggest challenges in defining the segments’ local CS is associated with the socket, which is inherently characterised by shape variability. Unlike rigid components such as screws, which are physically solid with it and universally identifiable, the socket system exhibits flexibility and variability that is needed to comfortably accommodate the residual limb, which is subject specific. This complicates consistency among measurements, therefore hindering standardization across studies but also affecting the reliability of *in-vitro* tests for socket mechanical characterization. The definitions given in this manuscript can be applicable on different socket shapes, as demonstrated by applying the same socket coordinate system definition to both PTFAs and PTTAs.

Another point of attention is the management of the static standing trial. It is worth noting that the transverse axis passing through the anterior iliac spine is usually not horizontal, as the prosthetic side is higher than the sound side, as part of standard RSP design practice. Depending on how the data collected during this phase of the procedure is to be used, different choices can be made. Specifically, if the static trial is intended for point calibration only, instructing the athlete to stand as still as possible within the capture volume will suffice for research purposes. However, as in the present paper, if the researcher is interested in capturing the RPF deformation during the running cycle, i.e. with the latter unloaded, one should request that the athlete stand and lean their body weight onto the sound side only. Since static kinematics reflect the athlete’s actual capabilities during the standing trial, both procedures for collecting static standing data will result in angles not being considered “neutral” for the pelvic segment and hip and knee joints. If the neutral asset is sought to help understand the actual motion around the neutral position, the standing trial should be collected with the transverse axis passing through the horizontal anterior iliac spine and the prosthetic knee fully extended. In this case, however, it is impossible to achieve an unloaded RPF. This further emphasises the idea that kinematics is highly subject-specific and difficult to normalise, and static posture subtraction should be avoided.

Although being promising, the proposed methodology requires further validation to ensure its applicability across diverse populations and prosthetic designs. The needed validation steps include establishing the reliability and reproducibility of the obtained quantities across different measurement conditions. Ideally, further studies would be necessary to assess: (i) the test-retest reliability of the protocol to ensure consistent results under the same measurement conditions; (ii) the inter-subject reliability, to evaluate how the protocol performs across athletes with different biomechanical characteristics; (iii) the inter-operator reproducibility; and, possibly, (iv) the minimal detectable changes associated with different measurement conditions. However, the limited number of high-level athletes and their heterogeneity limit the feasibility of conducting a broad validation as would be required. Addressing these limitations in future work will be crucial for advancing the protocol’s applicability. Standardization efforts and validation studies will provide a more robust foundation for the biomechanical assessment of Paralympic athletes, hopefully allowing improvement in prosthetic design and athletic performance.

Instrumental to these validation aims, to facilitate scientists worldwide to test the protocol and extend its validation, the source codes and GUI of the Olympia software for kinematic calculations, and the configuration files required to define the model for any Paralympic athlete, are published alongside the manuscript. The open access sharing of these resources aims to foster a globally validated methodology, benefiting the entire research community and contributing to the advancement of this field.

Unlike conventional motion analysis studies, this work does not propose a comparison of the obtained kinematic curves with normative data. This limitation is primarily due to this study presenting the first explicitly defined model for analysing running biomechanics in Paralympic athletes, making direct comparisons with previous methodologies challenging and formally incorrect: i.e., different models would employ distinct reference system definitions, with no directly comparable kinematics. Additionally, the running biomechanics of amputee athletes exhibit fundamental differences from those of non-amputee athletes, further restricting the applicability of normative datasets.

In conclusion, this research proposes a standardized approach to study the biomechanics of running in Paralympic athletes, providing a foundation for possibly optimizing prosthetic design, enhancing athletic performance, and fostering global collaboration to advance the field.

## Data Availability

The datasets presented in this study can be found in online repositories. The names of the repository/repositories and accession number(s) can be found in the article/Supplementary Material.
