# Supplementary material for: A motion capture protocol for the kinematic analysis of transfemoral and transtibial sprinters
Source: Front Bioeng Biotechnol. 2025 Nov 4;13:1655295. doi: 10.3389/fbioe.2025.1655295 (PMC12623396; doi:10.3389/fbioe.2025.1655295)
Supplement: Supplementary file 2 [file Supplementaryfile1.pdf]

Supplementary Material

Summary

1 Supplementary images on the running specific prosthesis components 2
1.1 STL support files 3
2 Definitions of Segments, markers and coordinate systems for the unaffected side and the upper body 4
2.1 Head 4
2.2 Arm and forearm 4
2.3 Upper Trunk 5
2.4 Pelvis 7
2.5 Thigh – sound limb and transtibial amputees 7
2.6 Tibia – sound limb 8
2.7 Foot – sound limb 8
3 Socket axes definition 10
3.1 Terms and definitions 10
3.2 Procedure for axes determination 13
4 Summary of the whole marker-set 16
5 Cardan sequences for kinematics estimation 20
6 Complementary results on the example dataset 23
7 XML configuration files, MATLAB code to solve the model 25
7.1 Folder Hierarchy and Files 25
7.2 Taxonomy and Acquisition management 26
7.3 Model definition files 26
7.4 Example of complete configuration file for data processing 31
7.5 MATLAB code 32
7.6 Future update and development 32

1 Supplementary images on the running specific prosthesis components

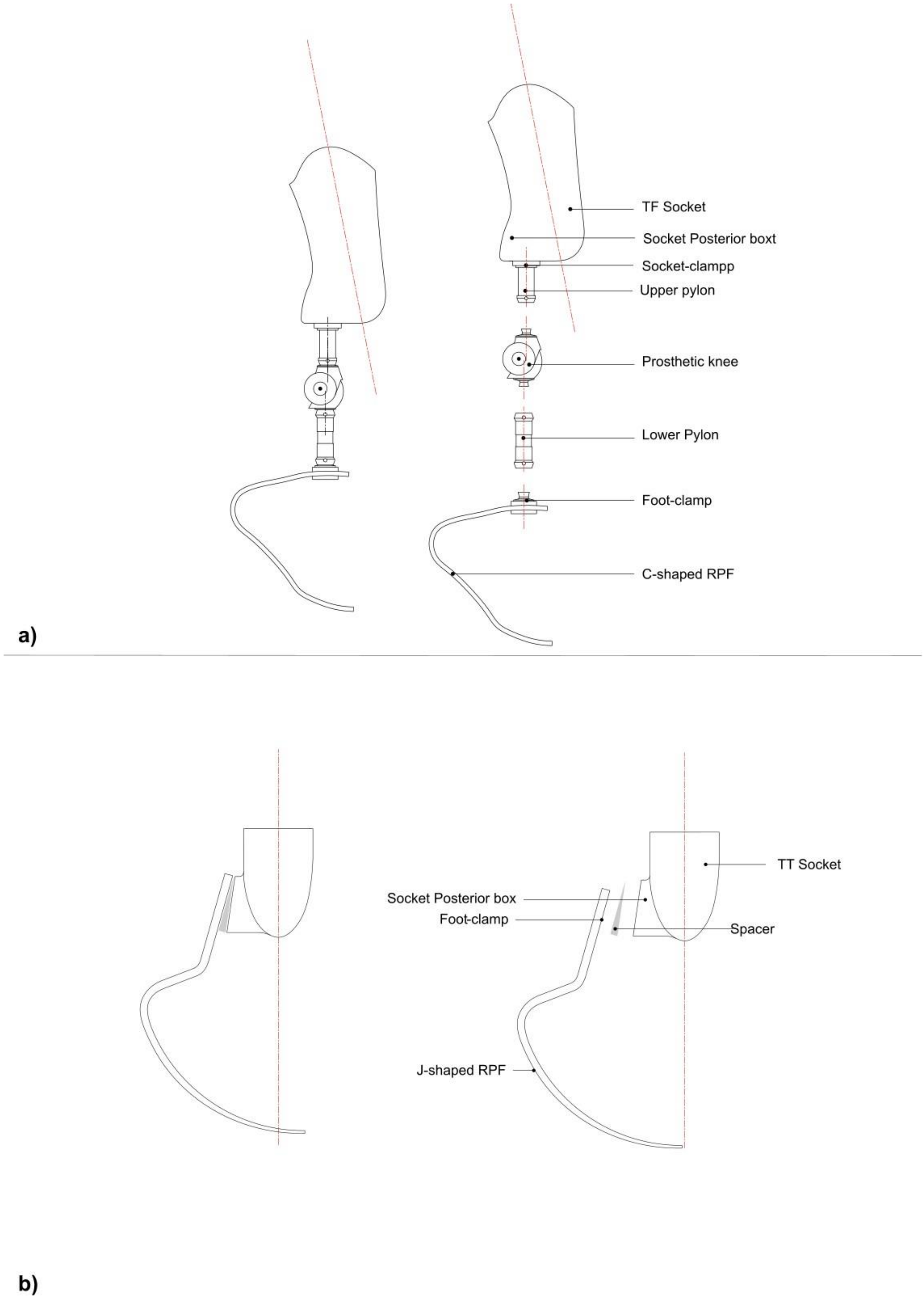

Supplementary Figure 1. Running Specific Prosthetic limb for people with transfemoral **(a)** and transtibial **(b)** amputations.

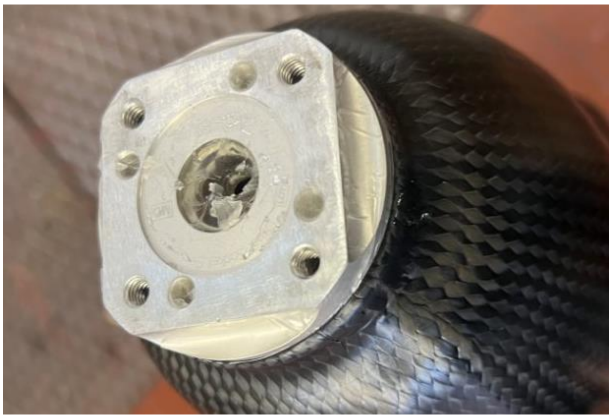

Supplementary Figure 2. Bottom view of a typical 4-screw socket-clamp adapter.

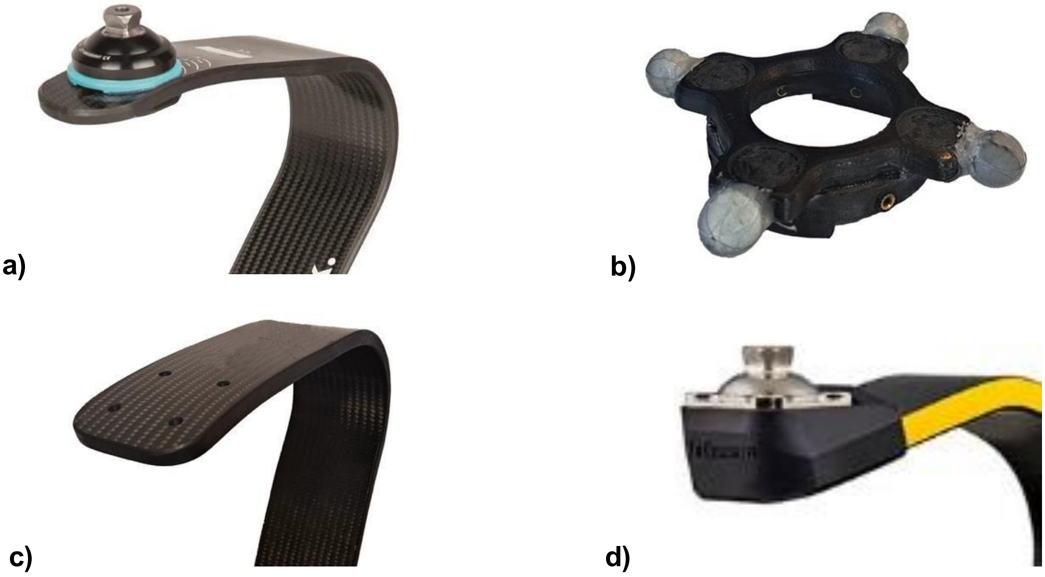

Supplementary Figure 3. (a) Running Prosthetic Foot 1E91 (Ottobock, Germany), with a sliding pyramid adapter foot-clamp. **(b)** Collar support for foot-clamp markers. **(c)** Running Prosthetic Foot 1E91 (Ottobock, Germany), with a foot-clamp fixed 4-hole pyramid adapter. **(d)** Running Prosthetic Foot Cheetah Xceed (Ossur, Iceland), with a foot-clamp fixed 5°-inclined 4-hole pyramid adapter.

1.1 STL support files

These Supplementary Materials are complemented with the following STL files:

- 3D printed crossed-shaped collar support (Supplementary Figure 3b): crossed-shaped_collar_support.STL

- 3D printed T-shaped support for the technical proximal RPF (main manuscript – Section 2.2.6.1): T-shaped_techRPF_support.STL

2 Definitions of Segments, markers and coordinate systems for the unaffected side and the upper body

Definitions for the upper body and non-prosthetic side of the lower body are given accordingly to the ISB recommendations (Wu et al., 2002) and the CAST protocol (Cappozzo et al., 1995).

In the following tables static and wand-calibrated markers are noted with S and W, respectively. Markers can be either anatomical (i.e., associated with anatomical landmarks), technical (i.e., normally used as cluster to reconstruct anatomical points and, thus, the Coordinate Systems), and mechanical (i.e., associated with the mechanical parts of the prostheses).

2.1 Head

Supplementary Table 1. List of markers for the head segment

Marker label	Marker type	Notes	Description
HF	Technical		Frontal
HR	Technical		Lateral right
HP	Technical		Posterior occipital extreme
HL	Technical		Lateral left
GB	Anatomical	S	Frontal bone glabella
<s>AM	Anatomical	W	Acoustic meatus
CH	Anatomical	S	Chin protuberance

The definition of the head coordinate system (abbreviated as *HD* and shown in Supplementary Figure 4a-b) is based on the points LAM, RAM, GB, CH.

Supplementary Table 2. Head Coordinate System definition

Axis/point	Formula	Description
Tag: *HD*		
${}^{G}O_{HD}$	$\frac{LAM + RAM}{2}$	Midpoint between LAM and RAM
${}^{G}\hat{y}_{HD}$	$\frac{GB - CH}{\|\|GB - CH\|\|}$	Line joining CH to GB
${}^{G}\hat{z}_{HD}$	$\frac{(GB - {}^{G}O_{HD}) \times {}^{G}\hat{y}_{HD}}{\|\|(GB - {}^{G}O_{HD}) \times {}^{G}\hat{y}_{HD}\|\|}$	Perpendicular to the quasi-sagittal plane defined by ${}^{G}\hat{y}_{HD}$ and the line connecting ${}^{G}O_{HD}$ to GB
${}^{G}\hat{x}_{HD}$	$\frac{{}^{G}\hat{y}_{HD} \times {}^{G}\hat{z}_{HD}}{\|\| {}^{G}\hat{y}_{HD} \times {}^{G}\hat{z}_{HD}\|\|}$	Perpendicular to the *yz* plane

2.2 Arm and forearm

Supplementary Table 3. List of markers for both arm and forearm segments

Marker	Marker type	Description
<s>AC	Anatomical	Acromion
<s>HLE	Anatomical	Humerus Lateral epicondyle
<s>MSS	Anatomical	Midpoint of the lateral and medial styloids of the forearm positioned on the dorsal aspect

The definition of the arm coordinate system (abbreviated as *AR* and shown in Supplementary Figure 4c) is based on the points AC, HLE, MSS.

Supplementary Table 4. Arm Coordinate System definition

Axis/point	Formula	Description
Tag: <s>AR		
${}^{G}O_{AR}$	HLE	Coincident with HLE
${}^{G}\hat{y}_{AR}$	$\frac{AC - HLE}{\|\|AC - HLE\|\|}$	Directed from HLE to AC
${}^{G}\hat{z}_{AR}$	$\frac{(MSS - HLE) \times {}^{G}\hat{y}_{AR}}{\|\|(MSS - HLE) \times {}^{G}\hat{y}_{AR}\|\|}$	Perpendicular to the plane defined by the vector directed from HLE to MSS and ${}^{G}\hat{y}_{AR}$ (to be adapted for left and right sides – this formula is for right side)
${}^{G}\hat{x}_{AR}$	${}^{G}\hat{y}_{AR} \times {}^{G}\hat{z}_{AR}$	Perpendicular to the *yz* plane

The definition of the forearm coordinate system (abbreviated as *FA* and shown in Supplementary Figure 4d) is based on the points AC, HLE, MSS.

Supplementary Table 5. Forearm Coordinate System definition

Axis/point	Formula	Description
Tag: <s>FA		
${}^{G}O_{FA}$	HLE	Coincident with HLE
${}^{G}\hat{y}_{FA}$	$\frac{HLE - MSS}{\|\|HLE - MSS\|\|}$	Directed from MSS to HLE
${}^{G}\hat{z}_{FA}$	$\frac{(HLE - AC) \times {}^{G}\hat{y}_{FA}}{\|\|(HLE - AC) \times {}^{G}\hat{y}_{FA}\|\|}$	Perpendicular to the plane defined by the vector directed from AC to HLE and ${}^{G}\hat{y}_{FA}$
${}^{G}\hat{x}_{FA}$	${}^{G}\hat{y}_{FA} \times {}^{G}\hat{z}_{FA}$	Perpendicular to the *yz* plane

2.3 Upper Trunk

Supplementary Table 6. List of markers for the upper trunk segment

Marker	Marker type	Description
IJ	Anatomical	Jugular Notch
PX	Anatomical	Xiphoid Process
T8	Anatomical	8th Thoracic vertebra
C7	Anatomical	7th Cervical vertebra

The definition of the upper trunk coordinate system (abbreviated as *TK* and shown in Supplementary Figure 4e-f) is based on the points IJ, T8, PX, C7.

Supplementary Table 7. Upper Trunk Coordinate System definition

Axis/point	Formula	Description
Tag: TK		
${}^{G}O_{TK}$	IJ	Coincident with IJ
${}^{G}M_1$	$\frac{T8 + PX}{2}$	Midpoint between T8 and PX
${}^{G}M_2$	$\frac{IJ + C7}{2}$	Midpoint between IJ and C7
${}^{G}\hat{y}_{TK}$	$\frac{M2 - M1}{\|\|M2 - M1\|\|}$	Directed from M1 to M2
${}^{G}\hat{z}_{TK}$	$\frac{(IJ - C7) \times {}^{G}\hat{y}_{TK}}{\|\|(IJ - C7) \times {}^{G}\hat{y}_{TK}\|\|}$	Perpendicular to the *xy* plane, defined by the vector directed from C7 to IJ and ${}^{G}\hat{y}_{TK}$
${}^{G}\hat{x}_{TK}$	${}^{G}\hat{y}_{TK} \times {}^{G}\hat{z}_{TK}$	Perpendicular to the *yz* plane

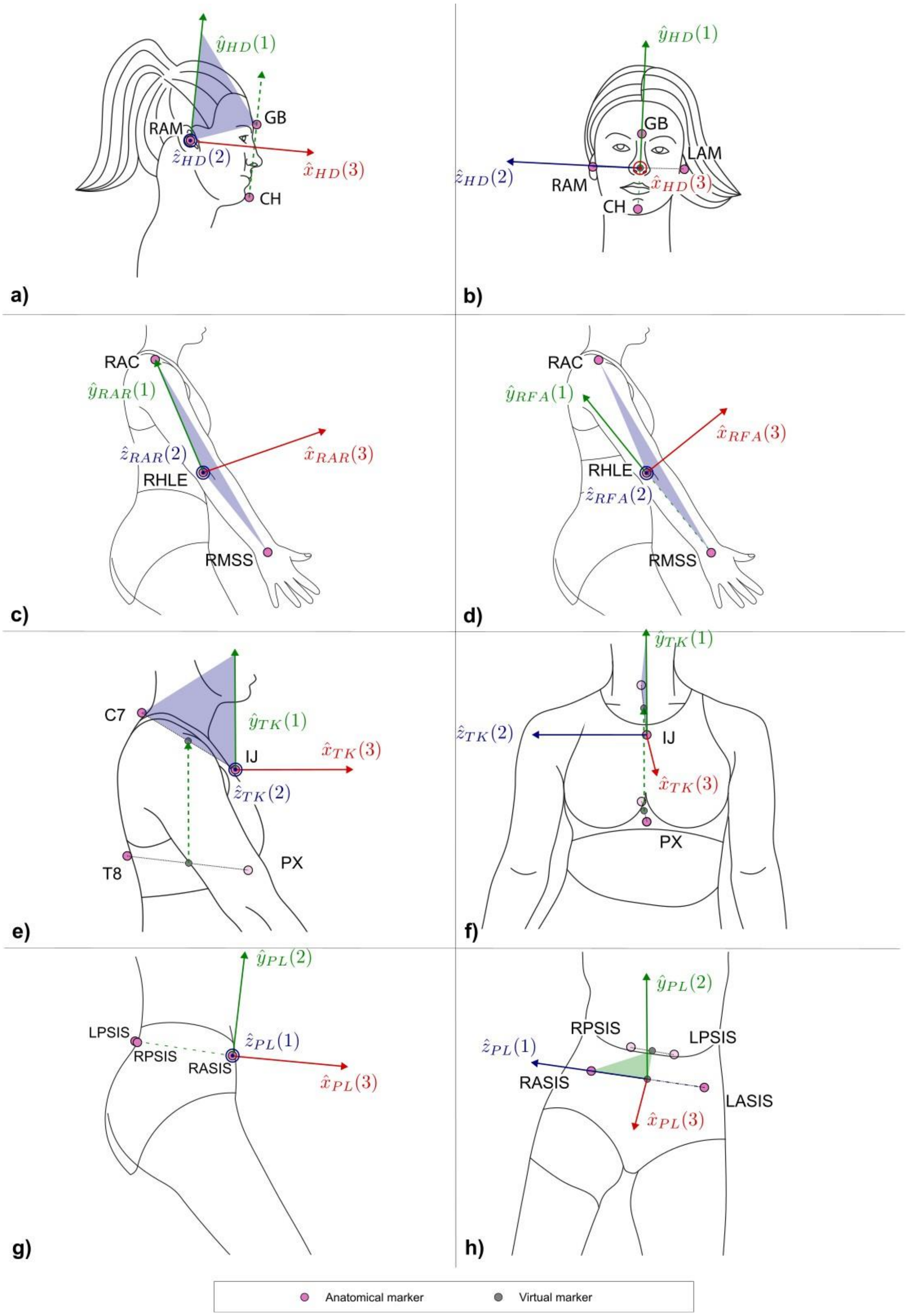

Supplementary Figure 4. Markers and the local coordinate system definitions of: **(a)** right lateral and **(b)** frontal views of the head; **(c)** lateral view of the right arm; **(d)** lateral view of the right forearm; **(e)** right lateral and **(f)** frontal views of the upper trunk; **(g)** right lateral and **(h)** frontal views of the pelvis. Light magenta markers are anatomical, while virtual calculated points are in grey. Numbers in round parentheses provide the order for the axis construction.

2.4 Pelvis

The pelvis segment is defined by the markers placed on the anterior and posterior iliac spines. The hip joint centres, needed to define the thigh local CS and, thus, the pelvis-thigh relative motion, are estimated over the pelvis local CS and according to statistical relationship over the anterior iliac spine distance (Bell et al., 1990, 1989).

Supplementary Table 8. List of markers for the pelvis segment

Marker	Marker type	Description
<s>ASIS	Anatomical	The most prominent aspect of the anterior superior iliac spine
<s>PSIS	Anatomical	The most prominent aspect of the posterior superior iliac spine
<s>HJC	Virtual	Estimated with (Bell et al., 1990)

The definition of the pelvis coordinate system (abbreviated as *FA* and shown in Supplementary Figure 4g-h) is based on the points LASIS, RASIS, LPSIS, RPSIS.

Supplementary Table 9. Pelvis Coordinate System definition

Axis/point	Formula	Description
Tag: *PL*		
${}^{G}O_{PL}$	$\frac{LASIS + RASIS}{2}$	Midpoint between LASIS and RASIS
$SACR$	$\frac{LPSIS + RPSIS}{2}$	Midpoint between LPSIS and RPSIS
${}^{G}\hat{z}_{PL}$	$\frac{RASIS - LASIS}{\lVert RASIS - LASIS \rVert}$	Directed from LASIS to RASIS
${}^{G}\hat{y}_{PL}$	$\frac{{}^{G}\hat{z}_{PL} \times ({}^{G}O_{PL} - SACR)}{\lVert {}^{G}\hat{z}_{PL} \times ({}^{G}O_{PL} - SACR) \rVert}$	Perpendicular to the plane defined by LASIS, RASIS and SACR, pointing upward
${}^{G}\hat{x}_{PL}$	${}^{G}\hat{y}_{PL} \times {}^{G}\hat{z}_{PL}$	Perpendicular to the *yz* plane

2.5 Thigh – sound limb and transtibial amputees

Supplementary Table 10. List of markers for the thigh segment

Marker	Marker type	Notes	Description
<s>GT	Anatomical		Greater Trochanter
<s>FLE	Anatomical	S if TT	Femur lateral epicondyle. Ask subject to slightly flex/extend the knee. <s>FLE is placed ideally on the sagittal trace in center of rotation of the knee joint.
<s>FME	Anatomical	S	Femur medial epicondyle. Placed following same methodology adopted for <s>FLE.
<s>HJC	Virtual		As defined in Pelvis CS (§2.4)
<s>T1	Technical		Given the lateral axis of the thigh, the marker is placed proximally (1/3 of GT-FLE) anterior (2 fingers) to the lateral axis.
<s>T2	Technical		Given the lateral axis of the thigh, the marker is placed distally (2/3 of GT-FLE) anterior (2 fingers) to the lateral axis.
<s>T3	Technical		Given the lateral axis of the thigh, the marker is placed proximally (1/3 of GT-FLE) posterior (2 fingers) to the lateral axis.
<s>T4	Technical		Given the lateral axis of the thigh, the marker is placed distally (2/3 of GT-FLE) posterior (2 fingers) to the lateral axis.

The lateral axis of the thigh is defined as the line connecting the great trochanter (GT) with the lateral epicondyle of the femur (FLE). In the case of transtibial amputation, with the socket covering the epicondyles, both knee epicondyles will be static markers placed by palpation before wearing the transtibial socket and associated with the thigh cluster.

The technical markers T1, T2, T3 and T4 are used to reconstruct static markers and possibly the GT, which may be covered by or lost for hand swinging during sports exercises.

The definition of the thigh coordinate system (abbreviated as *TH* and shown in Supplementary Figure 5a) is based on the points HJC, FLE, FME.

Supplementary Table 11. Thigh Coordinate System definition

Axis/point	Formula	Description
**Tag:** *<s>TH*		
${}^{G}O_{TH}$	$\frac{FLE+FME}{2}$	Midpoint between FLE and FME, which is the knee joint centre (KJC)
${}^{G}\hat{y}_{TH}$	$\frac{HJC-{}^{G}O_{TH}}{\lVert HJC-{}^{G}O_{TH}\rVert}$	Longitudinal axis, joining the ${}^{G}O_{TH}$ to the HJC
${}^{G}\hat{x}_{TH}$	$\frac{{}^{G}\hat{y}_{TH}\times(FLE-FME)}{\lVert {}^{G}\hat{y}_{TH}\times(FLE-FME)\rVert}$	Perpendicular to the plane defined by HJC, FLE, and FME, pointing forward (to be adapted for left and right sides – this formula is for right side)
${}^{G}\hat{z}_{TH}$	${}^{G}\hat{x}_{TH}\times{}^{G}\hat{y}_{TH}$	Perpendicular to the *xy* plane, pointing rightward

2.6 Tibia – sound limb

Supplementary Table 12. List of markers for the tibia segment

Marker	Marker type	Notes	Description
<s>TT	Anatomical		Tibial Tuberosity (most prominent point)
<s>HF	Anatomical		Head of Fibula (most prominent point)
<s>LM	Anatomical		Lateral Malleolus (most prominent point)
<s>MM	Anatomical	S	Medial Malleolus (most prominent point)
<s>SH1	Technical		Technical marker on the mid shank along the anterior aspect

The definition of the tibia coordinate system (abbreviated as *TB* and shown in Supplementary Figure 5b) is based on the points HF, LM, MM, TT.

Supplementary Table 13. Tibia Coordinate System definition

Axis/point	Formula	Description
**Tag:** *<s>TB*		
${}^{G}O_{TB}$	$\frac{LM+MM}{2}$	Midpoint between LM and MM, which is the ankle joint centre (KJC)
${}^{G}\hat{x}_{TB}$	$\frac{(HF-{}^{G}O_{TB})\times(LM-MM)}{\lVert(HF-{}^{G}O_{TB})\times(LM-MM)\rVert}$	Perpendicular to the quasi-frontal plane defined by LM, MM and HF, pointing forward (to be adapted for left and right sides – this formula is for right side)
${}^{G}\hat{z}_{TB}$	$\frac{{}^{G}\hat{x}_{TB}\times(TT-{}^{G}O_{TB})}{\lVert {}^{G}\hat{x}_{TB}\times(TT-{}^{G}O_{TB})\rVert}$	Perpendicular to the quasi-sagittal plane defined by ${}^{G}\hat{x}_{TB}$ and the line joining the ${}^{G}O_{TB}$ to TT, pointing rightward (to be adapted for left and right sides – this formula is for right side)
${}^{G}\hat{y}_{TB}$	${}^{G}\hat{z}_{TB}\times{}^{G}\hat{x}_{TB}$	Perpendicular to the *zx* plane

2.7 Foot – sound limb

Supplementary Table 14. List of markers for the foot segment

Marker	Marker type	Notes	Description
<s>CA	Anatomical		Posterior aspect of calcaneus, just below the Achille's tendon insertion
<s>IMH	Anatomical	S	First metatarsal distal head
<s>IIMH	Anatomical		Second metatarsal distal head
<s>VMH	Anatomical		Fifth metatarsal distal head
<s>F1	Technical		On the lateral side of the shoe, ideally at equally distanced to CA and IIMH, but not aligned to them

The definition of the thigh coordinate system (abbreviated as *FT* and shown in Supplementary Figure 5c) is based on the points CA, IMH, IIMH, VMH.

Supplementary Table 15. Foot Coordinate System definition

Axis/point	Formula	Description
**Tag:** *<s>FT*		
${}^{G}O_{FT}$	CA	Coincident to CA
${}^{G}\hat{y}_{FT}$	$\frac{(VMH - CA) \times (IMH - CA)}{\lVert(VMH - CA) \times (IMH - CA)\rVert}$	Perpendicular to the plane defined by IMH, VMH and CA pointing upward (to be adapted for left and right sides – this formula is for right foot)
${}^{G}\hat{z}_{FT}$	$\frac{(IIMH - CA) \times {}^{G}\hat{y}_{FT}}{\lVert(IIMH - CA) \times {}^{G}\hat{y}_{FT}\rVert}$	Perpendicular to the plane defined by ${}^{G}\hat{y}_{FT}$ and IIMH, pointing rightward (to be adapted for left and right sides – this formula is for right foot)
${}^{G}\hat{x}_{FT}$	${}^{G}\hat{y}_{FT} \times {}^{G}\hat{z}_{FT}$	Perpendicular to the *yz* plane

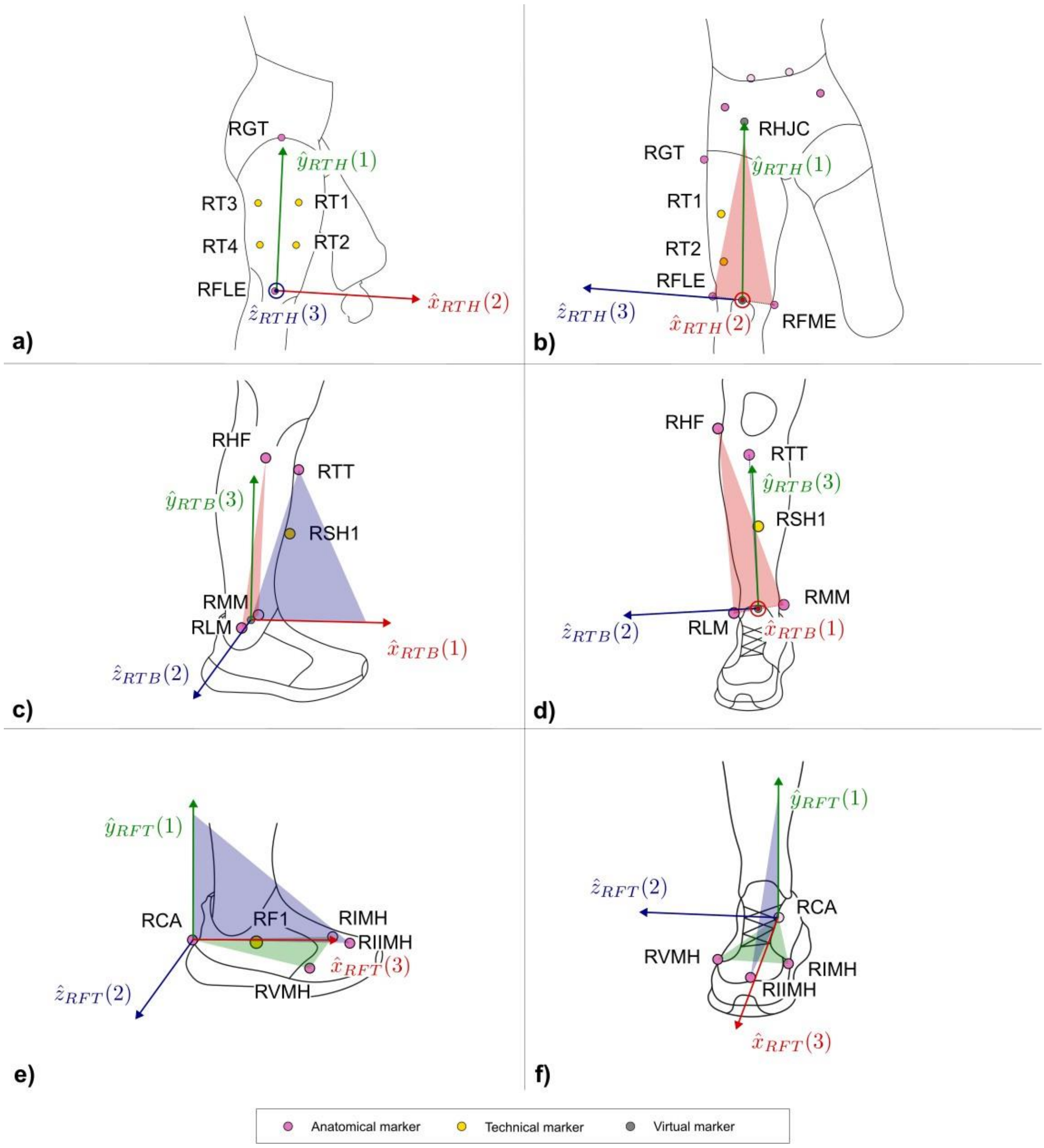

Supplementary Figure 5. Markers and the local coordinate system definitions of: **(a)** right lateral and **(b)** frontal views of the thigh; **(c)** right lateral and **(d)** frontal view of the tibia; **(e)** right lateral and **(f)** frontal view of the foot. Light magenta markers are anatomical, yellow markers are technical, while virtual calculated points are in grey. Numbers in round parentheses provide the order for the axis construction.

3 Socket axes definition

This section defines terms and procedures to define the axes of the prosthetic socket of a person with either transfemoral or transtibial amputation. The procedure assumes the user of the socket is present.

3.1 Terms and definitions

The following sections use the terms and definitions given below and those provided in the ISO 8548-2[1], ISO 8549-1[2], ISO 8549-2[3], ISO 13405-1[4], ISO 29783-3[5], ISO 10328[6], ISO 22675[7].

Term	Definition
**plumb line**	line parallel to the gravity vector
**standard anatomical posture**	the body is erect with the head and torso upright; the arms are at the sides of the torso with the shoulders in neutral rotation, elbows extended, the cubital fossae of the elbow and the palms face forward, the fingers are extended, and the thumbs are adducted with the pad of each thumb facing forward. The lower extremities are straight and parallel, with the second toe facing straight forward.[8]
**standard anatomical posture coordinate system**<br>**body coordinate system**	coordinate system formed by the intersection of the sagittal, frontal and transverse anatomical planes when the person is in the standard anatomical posture, with the upward axis called *u*, the medio-lateral axis called *o* and the anterior-posterior axis called *f*, positive in the direction of progression. The origin is set at the intersection of the three anatomical planes, when the transverse plane overlaps the floor.

[1] ISO 8548-2:2020, Prosthetics and orthotics — Limb deficiencies - Part 2: Method of describing lower limb amputation stumps

[2] ISO 8549-1, Prosthetics and orthotics — Vocabulary — Part 1: General terms for external limb prostheses and external orthoses

[3] ISO 8549-2, Prosthetics and orthotics — Vocabulary — Part 2: Terms relating to external limb prostheses and wearers of these prostheses

[4] ISO 13405-1:2014, Prosthetics and orthotics — Classification and description of prosthetic components — Part 1: Classification of prosthetic components

[5] ISO 29783-3:2016, Prosthetics and orthotics — Vocabulary Part 1: Normal gait

[6] ISO 10328:2016, Prosthetics — Structural testing of lower-limb prostheses — Requirements and test methods

[7] ISO 22675:2016, Prosthetics — Testing of ankle-foot devices and foot units — Requirements and test methods

[8] Selinger A, Physical Rehabilitation, Chapter 4 - Posture

Term	Definition
**prosthetic alignment reference posture**<br>**reference posture**	posture of the person with amputation standing upright with the head, trunk and pelvis medio-lateral and anterior-posterior axes parallel to the *f*- and *o*- axes, the residual limb ankle center under the ASIS, equal distance from the most medial part of the limb and the anatomical sagittal plane, the socket donned with the distal part resting on a stood with non-slippery top, body weight equally distributed on the right and left side.<br><br>*TF specifications:* the medial brim of the socket will set the internal-external rotation of the hip.<br><br>*TT specifications:* the MPT-PF axis laying on a plane parallel to the sagittal anatomical plane. If the foot of the prosthesis is mounted on the socket, there is no need to use the stood. If the prosthesis is higher than the contralateral limb, e.g. in case of a sport prosthesis, height compensation under the prosthetic foot is needed to ensure that the pelvis is parallel to the *o*-axis.[9,10,11]
**line of progression**	imaginary direction that the body follows during forward movement
**posterior shelf**	proximal posterior area of a transtibial socket
**50:50 gauge**	planar pantograph gauge with a perpendicular rod positioned at each extreme and a central hole dividing the distance between the rods in half.[12]
**height-adjustable crossline laser**<br>**laser**	laser system, standing on a high-adjustable tripod, projecting a set of cross lines aligned with gravity or perpendicular to it, and a second laser line quick can be manually angled relative to gravity.
**mechanical line of the lower limb**<br>**mechanical axis of the lower limb**	in the frontal anatomical plane, line passing through the hip joint centre and the ankle joint centre when the person is in the reference posture.[13]

[9] the specification about the MPT-PF line is important to standardize hip rotation.

[10] In this position, the posterior side of the knee should lay in the frontal plane, i.e. the posterior shelf should be facing almost perpendicular to *f* or slightly internally rotated. This is due to the tibial tubercle (TB) being lateral to the femur trochlear grove (TG) (Shu et al., 2020). However, MPT is medial to TB, which should decrease the difference. The MPT-PF line is preferred as reference landmark compared to the line of posterior shelf because of the complex shape this latter can assumed, which is dictated by the dynamic motion of the shank relative to the femur during knee flexion.

[11] In case of a persons with bilateral transtibial amputation the upright posture cannot be maintained. In this case, the subject is assumed to lay on an assessment flat bed, with the frontal plane replaced by the transverse plane.

[12] See Ottobock 743A80.

[13] In prosthetics, where radiographic imaging is not available, the proximal point is not the hip joint centre but the anterior superior iliac spine (ASIS).

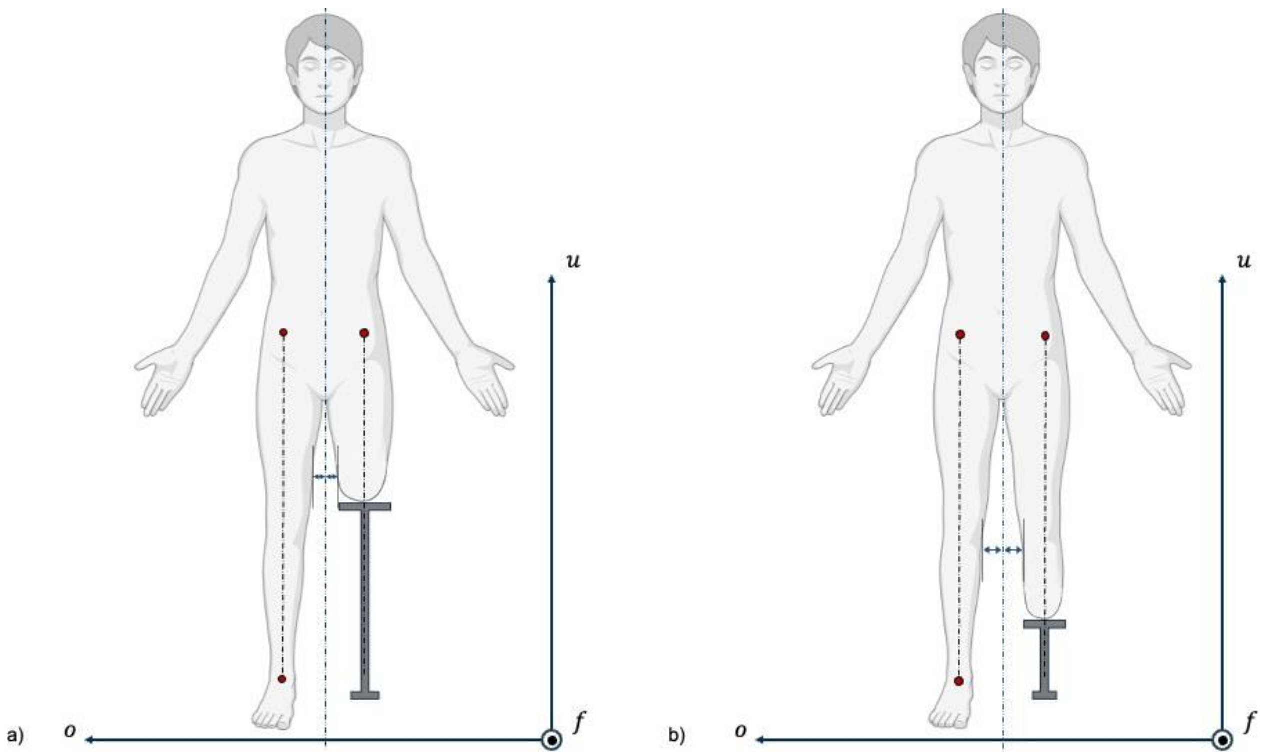

Supplementary Figure 6. Prosthetic alignment reference posture for people with **(a)** transfemoral and **(b)** transtibial amputation.

The Supplementary Table 16 provides the definitions of the relevant notable points needed for the procedure.

Supplementary Table 16. Symbols, definitions and estimation

Symbol	Definition	Estimation
u-axis	Upward axis of the body CS	Intersection of the frontal and sagittal anatomical planes
f-axis	Forward axis of the body CS	Intersection of the frontal and transverse anatomical planes
o-axis	Outward (lateral) axis of the body CS	Intersection of the frontal and sagittal anatomical planes
M2S	Sartorius flexor tendon	Most medial point of the Sartorius flexor tendon
A0	Most distal point of the socket	Most distal and apical point of the socket shape, assuming the socket not comprising the distal connection (typical of modular prostheses)
MPT	Mid Patellar Tendon	Central point of the tendon<br>(for transtibial amputation only)
PF	Popliteal Fossa<br>Popliteal Depression	Center of the diamond-shaped anatomical space located at the posterior aspect of the knee joint. It can be estimated as the point of application of a force pointing forward opposing a force applied on the MPT pointing backward<br>(for transtibial amputation only)

Symbol	Definition	Estimation
LA	Lateral axis of the socket	Axis passing through LAP and LAD, oriented proximally
FA	Frontal axis of the socket	Axis passing through FAP and FAD, oriented proximally
AJC	Ankle joint center	Midpoint of the medial and lateral malleoli
mA	Mechanical axis of the lower-limb	Considering the contralateral side, vertical line passing through the ASIS and the AJC when the person is in the reference posture, oriented proximally

3.2 Procedure for axes determination

The subject using the prosthesis is assumed to be available, with the socket donned and to be in the reference alignment posture. Then the axes are marked on the socket by the certified prosthetist or other technical staff. Procedures are described below and shown in Supplementary Figure 7-8.

3.2.1 Transfemoral socket

The certified prosthetist, holding the 50:50 gauge in the sagittal plane (lateral side) of the body:

1. measures the distance between A0 and GT;
2. divides the distance A0-GT in 3 parts and marks the LP and LD points on the A0-GT line;
3. closes the gauge until the rods touch the sides of the socket moving on the proximal third of the distance A0-GT (i.e., at the LP height);
4. marks the midpoint between the rods taking advantage of the gauge central hole defining LAP;
5. closes the gauge until the rods touch the sides of the socket moving on the distal third of the distance A0-GT (i.e., at the LD height);
6. marks the midpoint between the rods taking advantage of the gauge central hole defining LAD;
7. draws the "Lateral Axis of the Socket" as the line connecting LAP and LAD.

The certified prosthetist moves the laser in the frontal plane of the body and then:

8. repeat points 1) to 6) substituting the distance A0-GT with the A0-M2S to define AP and AD, FAP and FAD;
9. draws the "Frontal Axis of the Socket" as the line connecting FAP and FAD;
10. an additional point (MAD) is estimated on the medial side of the socket as symmetrical to LAD with respect to the LAP-LAD axis.

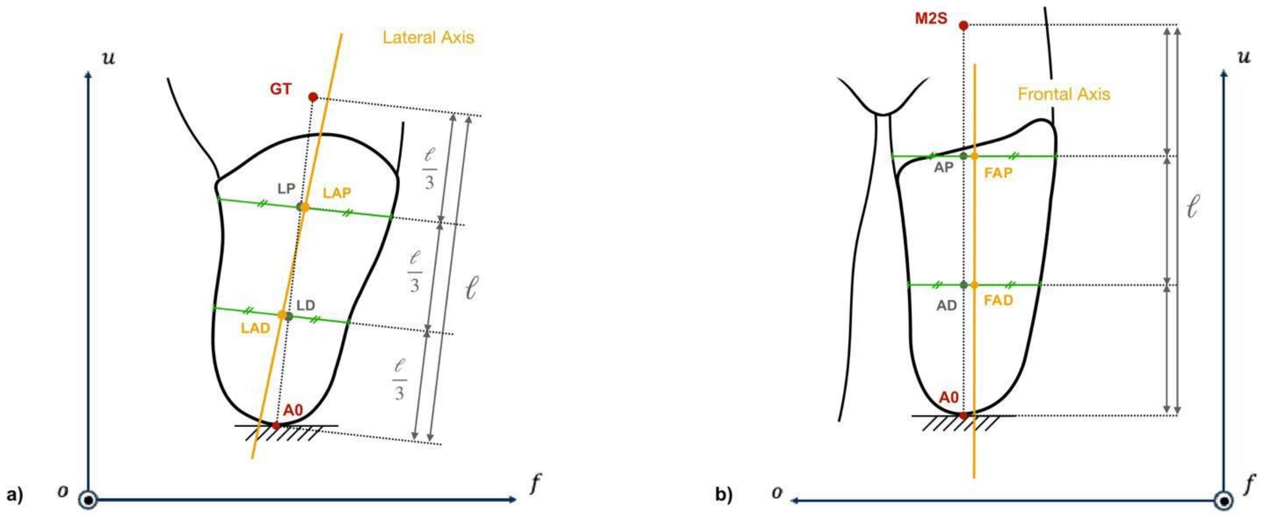

Supplementary Figure 7. Procedure to draw the **(a)** lateral and **(b)** frontal axes of the socket for people with transfemoral amputation.

3.2.2 Transtibial socket

The certified prosthetist, holding the 50:50 gauge in the sagittal plane (lateral side) of the body:

1. touches the socket at MPT and PF;
2. closes the gauge until the rods touch the sides of the socket;
3. marks the midpoint between the rods taking advantage of the gauge central hole. This landmark is the “Lateral Axis Proximal” (LAP);
4. repeats points 1) and 2) at a point 40 mm above the distal end of the socket;
5. adjusts the horizontal inclination of the gauge while closing it further, until the rods reach the minimum distance;
6. marks the midpoint between the rods taking advantage of the gauge central hole. This point is the “Lateral Axis Distal” (LAD);
7. draws the line connecting LAP and LAD. This is the “Lateral Axis of the Socket” (LA).

The certified prosthetist moves the laser in the frontal plane of the body CS and then:

8. with a 50:50 gauge touches the socket at 25 mm below the MPT;
9. closes the gauge until the rods touch the sides of the socket;
10. adjusts the horizontal inclination of the gauge while closing it further, until the rods reach the minimum distance;
11. repeat point 3 and 7. The landmarks are called “Frontal Axis Proximal” (FAP) and “Frontal Axis Distal” (FAD);
12. the line between FAP and FAD is the frontal axis of the socket (FA);
13. an additional point is estimated on the medial side of the socket as symmetrical to LAD with respect to the MPT-PF axis. This point is called Medial Axis Proximal (MAD).

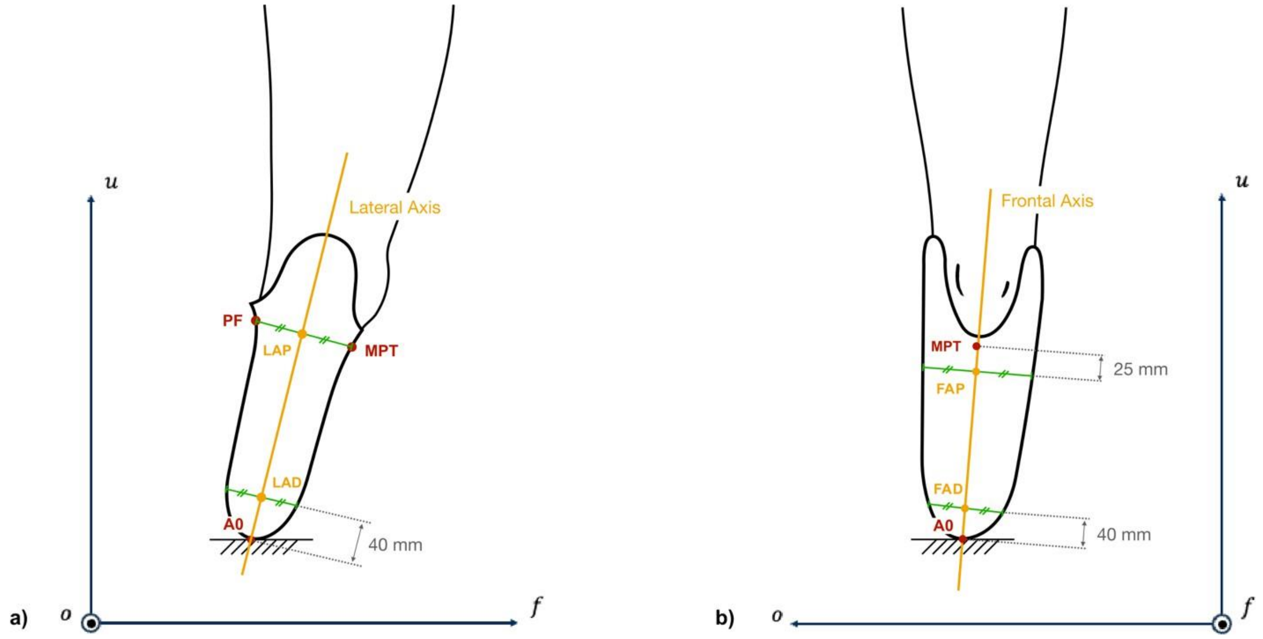

Supplementary Figure 8. Procedure to draw the **(a)** lateral and **(b)** frontal axes of the socket for people with transfemoral amputation.

4 Summary of the whole marker-set

Supplementary Table 17. List of markers grouped by body segment or mechanical part. Marker type indicates whether the marker is: (A) anatomical, referred to a specific anatomical landmark; (T) technical, either to build a technical cluster to be used as a reference to calibrate anatomical landmarks position or non-specifically associated with anatomical positions, which are needed to define the local coordinate systems; and (M) mechanical, referred to specific mechanical parts, or aligned with axes of mechanical parts. When virtual, referred to the inner of body parts or joints, a V precedes the A/M type. The column “Notes” reports additional information on marker type depending on the considered configuration of the acquisition setup. Notes can be: (S) static markers, those to be placed for static standing and to be removed during dynamic tasks; (W) wand calibrated, for those to be calibrated using a pointer equipped with markers with respect to a cluster (Cappozzo et al., 1995).

Group	Marker name	Marker type	Notes	Description
**Head**	HF	T		Frontal
	HR	T		Lateral right
	HP	T		Posterior occipital extreme
	HL	T		Lateral left
	GB	A	S	Frontal bone glabella
	<s>AM	A	W	Acoustic meatus
	CH	A	S	Chin protuberance
**Trunk**	IJ	A		Jugular Notch
	PX	A	S	Xiphoid Process
	T8	A	S	8th Thoracic vertebra
	C7	A	S	7th Cervical vertebra
**Arm**	<s>AC	A		Acromion
	<s>HLE	A		Humerus Lateral epicondyle
	<s>MSS	A		Midpoint of the lateral and medial styloids of the forearm positioned on the dorsal aspect
**Pelvis**	<s>ASIS	A		The most prominent aspect of the anterior superior iliac spine
	<s>PSIS	A	S	The most prominent aspect of the posterior superior iliac spine
	<s>HJC	VA		Estimated with (Bell et al., 1990, 1989)
**Thigh (UL/TT)**	<s>GT	A		Greater Trochanter

Group	Marker name	Marker type	Notes	Description
	<s>FLE	A	S if TT[14]	Femur lateral epicondyle. Ask subject to slightly flex/extend the knee. LE is placed ideally on the sagittal trace in centre of rotation of the knee joint.
	<s>FME	A	S	Femur medial epicondyle. Placed following same methodology adopted for <s>FLE.
	<s>T1	T		The marker is placed proximally (1/3 of GT-FLE) anterior (2 fingers) to the lateral axis[15].
	<s>T2	T		The marker is placed distally (2/3 of GT-FLE) anterior (2 fingers) to the lateral axis[2].
	<s>T3	T		The marker is placed proximally (1/3 of GT-FLE) posterior (2 fingers) to the lateral axis[2].
	<s>T4	T		The marker is placed distally (2/3 of GT-FLE) posterior (2 fingers) to the lateral axis[2].
**Tibia (UL)**	<s>TT	A		Tibial Tuberosity
	<s>HF	A		Head of Fibula
	<s>LM	A		Lateral Malleolus
	<s>MM	A	S	Medial Malleolus
	<s>SH1	T		Technical marker on the mid shank along the anterior aspect
**Foot (UL)**	<s>CA	A		Posterior aspect of calcaneus, just below the Achille's tendon insertion
	<s>IMH	A	S	First metatarsal distal head
	<s>IIMH	A		Second metatarsal distal head
	<s>VMH	A		Fifth metatarsal distal head
	<s>F1	T		On the midpoint of the lateral side of the shoe
**Socket**	<s>FAP	T		Proximally (1/4 of the socket length) on the frontal axis[16] of the socket
	<s>FAD	T		Distally (3/4 of the socket length) on the frontal axis[3] of the socket
	<s>LAP	T		Proximally (1/4 of the socket length) on the lateral axis[17] of the socket
	<s>LAD	T		Distally (3/4 of the socket length) on the lateral axis[4] of the socket

[14] For transtibial amputees, in the case of knee epicondyles being covered by the socket, both knee epicondyles markers will be static markers placed by analogy on the contralateral limb.

[15] The thigh lateral axis connects the Great Trochanter (GT) to the femoral lateral epicondyle (FLE).

[16] The socket frontal axis is the line joining the midpoints of the basis of the isosceles trapezium approximating the socket in the frontal plane as proposed in (Migliore et al., 2021) – see also section 3.

[17] The socket lateral axis is the line joining the midpoints of the basis of the isosceles trapezium approximating the socket in the lateral plane as proposed in (Migliore et al., 2021) – see also section 3.

Group	Marker name	Marker type	Notes	Description
	<s>PAD	T		Distally (3/4 of the socket length) on the posterior part of the socket, with no crucial alignment
	<s>MAD	T	S	Distally (3/4 of the socket length) on the medial axis of the socket
	<s>GT	A	TF only	Great trochanter
**Prosthetic knee**	<s>KL	M	TF only	Placed laterally on the prosthetic knee centre of rotation
	<s>KM	M	TF only	Placed medially on the prosthetic knee centre of rotation
**Socket-Clamp TF**	<s>WAM	M	W – TF only	Screw of the socket connector – anterior-medial
	<s>WAL	M	W – TF only	Screw of the socket connector – anterior-lateral
	<s>WPM	M	W – TF only	Screw of the socket connector – posterior-medial
	<s>WPL	M	W – TF only	Screw of the socket connector – posterior-lateral
**Foot-Clamp TF**	<s>CLL	M		Foot-Clamp: lateral
	<s>CLA	M		Foot-Clamp: anterior
	<s>CLM	M		Foot-Clamp: medial
	<s>CLP	M		Foot-Clamp: posterior
	<s>CLAL	M	W	Anterior lateral screw of the 4-hole foot-clamp
	<s>CLPL	M	W	Posterior lateral screw of the 4-hole foot-clamp
	<s>CLAM	M	W	Anterior medial screw of the 4-hole foot-clamp
	<s>CLPM	M	W	Posterior medial screw of the 4-hole foot-clamp
**Foot-Clamp TT**	<s>CLPL	M		Foot-Clamp: lateral proximally
	<s>CLDL	M		Foot-Clamp: lateral distally
	<s>CLPM	M		Foot-Clamp: medial proximally
	<s>CLDM	M		Foot-Clamp: medial distally
	<s>WP	M	W	Screw to connect the prosthetic foot to the socket – proximal screw
	<s>WD	M	W	Screw to connect the prosthetic foot to the socket – distal screw
**Proximal RPF**	<s>FPA	T	S if TF	Foot proximal anterior - Placed as anteriorly as possible on the mid-line of the prosthetic foot
	<s>FPA	T	TT only	Foot proximal anterior – Placed on the mid-line of the prosthetic foot two fingers below the clamp
	<s>FP1	T		Placed on a T-frame clamped to the prosthetic foot, proximally to clamp.
	<s>FP2	T		Placed on a T-frame clamped to the prosthetic foot, distally to clamp on the same line of FP1.
	<s>FP3	T		Placed on a T-frame clamped to the prosthetic foot, perpendicularly downwards to the line joining FP1 and FP2.

Group	Marker name	Marker type	Notes	Description
**Distal RPF**	<s>FDA	T		The most distal point (tip) of the prosthetic foot
	<s>FDM	T		Placed medially on the foot tip, 50 mm proximally from FDA
	<s>FDL	T		Placed laterally on the foot tip, 50 mm proximally from FDA
**Deformable group of markers**				
**Mid-RPF**	<s>FM1	T		Placed medially at the bending midpoint
	<s>FL1	T		Placed laterally at the bending midpoint
	<s>FM2	T		Placed in the midline of the foot at 1/3 of the distance between the FM1-FL1 midpoint and FDL
	<s>FM3	T		Placed in the midline of the foot at 2/3 of the distance between the FM1-FL1 midpoint and FDL

Markers pertaining to the deformable group are not to be intended for coordinate system construction, but rather for following the running prosthetic foot deformation during stance, so to inform or validate in-vitro simulations.

5 Cardan sequences for kinematics estimation

Supplementary Table 18. Joint Coordinate System and relevant Cardan sequences for the anatomical and mechanical joints relative rotations.

Joint name	Joint axis	Segment axis	Rotation
**Neck**	*Proximal segment:* Trunk – *TK*		
(tag: *n*)	*Distal segment:* Head – *HD*		
	$\hat{e}_1$	$\hat{z}_{TK}$	Flexion (—) / Extension (+)
	$\hat{e}_2$	$\hat{e}_3 \times \hat{e}_1$	Lateral flexion (right: +; left: —)
	$\hat{e}_3$	$\hat{y}_{HD}$	Lateral rotation (right: —; left: +)
	Cardan sequence: zx'y''		
**Elbow**	*Proximal segment:* Arm – *AR*		
(tag: *e*)	*Distal segment:* Forearm – *FA*		
	$\hat{e}_1$	$\hat{z}_{AR}$	Flexion (+) / Extension (—)
	$\hat{e}_2$	$\hat{e}_3 \times \hat{e}_1$	—
	$\hat{e}_3$	$\hat{y}_{FA}$	—
	Cardan sequence: zx'y'' (only first rotation should be considered)		
**Lumbosacral joint**	*Proximal segment:* Pelvis – *PL*		
(tag: *pt*)	*Distal segment:* Trunk – *TK*		
	$\hat{e}_1$	$\hat{z}_{PL}$	Flexion (—) / Extension (+)
	$\hat{e}_2$	$\hat{e}_3 \times \hat{e}_1$	Lateral flexion (right: +; left: —)
	$\hat{e}_3$	$\hat{y}_{TK}$	Lateral rotation (right: —; left: +)
	Cardan sequence: zx'y''		
**Hip**	*Proximal segment:* Pelvis – *PL*		
(tag: *h*)	*Distal segment:* Thigh – *TH*		
	$\hat{e}_1$	$\hat{z}_{PL}$	Flexion (+) / Extension (—)
	$\hat{e}_2$	$\hat{e}_3 \times \hat{e}_1$	Abduction (right: —; left: +) / Adduction (right: +; left: —)
	$\hat{e}_3$	$\hat{y}_{TH}$	Internal (right: +; left: —) / External (right: —; left: +) rotation
	Cardan sequence: zx'y''		
**Socket-pelvis in TF**	*Proximal segment:* Pelvis – *PL*		
(tag: *htf*)	*Distal segment:* Socket-TF – *SK*		
	$\hat{e}_1$	$\hat{z}_{PL}$	Flexion (+) / Extension (—)
	$\hat{e}_2$	$\hat{e}_3 \times \hat{e}_1$	Abduction (right: —; left: +) / Adduction (right: +; left: —)
	$\hat{e}_3$	$\hat{y}_{SK}$	Internal (right: +; left: —) / External (right: —; left: +) rotation
	Cardan sequence: zx'y''		
**Knee**	*Proximal segment:* Thigh – *TH*		
(tag: *k*)	*Distal segment:* Tibia – *TB*		

Joint name	Joint axis	Segment axis	Rotation
	$\hat{e}_1$	$\hat{z}_{TH}$	Flexion (—) / Extension (+)
	$\hat{e}_2$	$\hat{e}_3 \times \hat{e}_1$	Abduction (right: —; left: +) / Adduction (right: +; left: —)
	$\hat{e}_3$	$\hat{y}_{TB}$	Internal (right: +; left: —) / External (right: —; left: +) rotation
	Cardan sequence: zx'y''		
**Prosthetic knee**	*Proximal segment:* Prosthetic Proximal Functional Knee System – *PK*		
(tag: *pk*)	*Distal segment:* Distal Functional Knee System – *DK*		
	$\hat{e}_1$	$\hat{z}_{PK}$	Flexion (—) / Extension (+)
	$\hat{e}_2$	$\hat{e}_3 \times \hat{e}_1$	—
	$\hat{e}_3$	$\hat{y}_{DK}$	—
	Cardan sequence: zx'y'' (only first rotation is considered, others are used as alignment quality check)		
**Socket-thigh in TT**	*Proximal segment:* Thigh – *TH*		
(tag: *ktt*)	*Distal segment:* Socket-TT – *SK*		
	$\hat{e}_1$	$\hat{z}_{TH}$	Flexion (—) / Extension (+)
	$\hat{e}_2$	$\hat{e}_3 \times \hat{e}_1$	Abduction (right: —; left: +) / Adduction (right: +; left: —)
	$\hat{e}_3$	$\hat{y}_{SK}$	Internal (right: +; left: —) / External (right: —; left: +) rotation
	Cardan sequence: zx'y''		
**Ankle**	*Proximal segment:* Tibia – *TB*		
(tag: *a*)	*Distal segment:* Foot – *FT*		
	$\hat{e}_1$	$\hat{z}_{TB}$	Plantarflexion (—) / Dorsiflexion (+)
	$\hat{e}_2$	$\hat{e}_3 \times \hat{e}_1$	Internal (right: —; left: +) / External (right: +; left: —) rotation
	$\hat{e}_3$	$\hat{x}_{FT}$	Inversion (right: +; left: —) / Eversion (right: —; left: +)
	Cardan sequence: zy'x''		
**Virtual ankle**	*Proximal segment:* Foot-Clamp – *FC*		
(tag: *va*)	*Distal segment:* Distal Prosthetic Foot – *FD*		
	$\hat{e}_1$	$\hat{z}_{FC}$	Plantarflexion (—) / Dorsiflexion (+)
	$\hat{e}_2$	$\hat{e}_3 \times \hat{e}_1$	Internal (right: —; left: +) / External (right: +; left: —) rotation
	$\hat{e}_3$	$\hat{x}_{FD}$	Inversion (right: +; left: —) / Eversion (right: —; left: +)
	Cardan sequence: zy'x''<br>*Linear motion: linear displacement of the CS_{FD} origin relative to CS_{FC}*		

Supplementary Table 19. Joint Coordinate System and relevant Cardan sequences to estimate the relative rotations between the equivalent prosthetic leg and the prosthetic foot.

Joint name	Joint axis	Segment axis	Rotation
**Distal RPF - GT-K**	*Proximal segment:* GT-K – *GK*		
	Distal segment: Distal Prosthetic Foot – *FD*		
	$\hat{e}_1$	$\hat{z}_{GT-K}$	Plantarflexion (—) / Dorsiflexion (+)
	$\hat{e}_2$	$\hat{e}_3 \times \hat{e}_1$	Internal (right: —; left: +) / External (right: +; left: —) rotation
	$\hat{e}_3$	$\hat{x}_{FD}$	Inversion (right: +; left: —) / Eversion (right: —; left: +)
	Cardan sequence: zy'x''<br>*Linear motion: linear displacement of the CS_{FD} origin relative to 2-Dimensional CS_{GT-K}. The Distal Foot Origin must be deprived of the non-sagittal component (i.e., projected on the sagittal plane).*		
**Distal RPF - HJC-K**	*Proximal segment:* HJC-K – *HK*		
	Distal segment: Distal Prosthetic Foot – *FD*		
	$\hat{e}_1$	$\hat{z}_{HJC-K}$	Plantarflexion (—) / Dorsiflexion (+)
	$\hat{e}_2$	$\hat{e}_3 \times \hat{e}_1$	Internal (right: —; left: +) / External (right: +; left: —) rotation
	$\hat{e}_3$	$\hat{x}_{FD}$	Inversion (right: +; left: —) / Eversion (right: —; left: +)
	Cardan sequence: zy'x''<br>*Linear motion: linear displacement of the CS_{FD} origin relative to 3-Dimensional CS_{HJC-K}*		

6 Complementary results on the example dataset

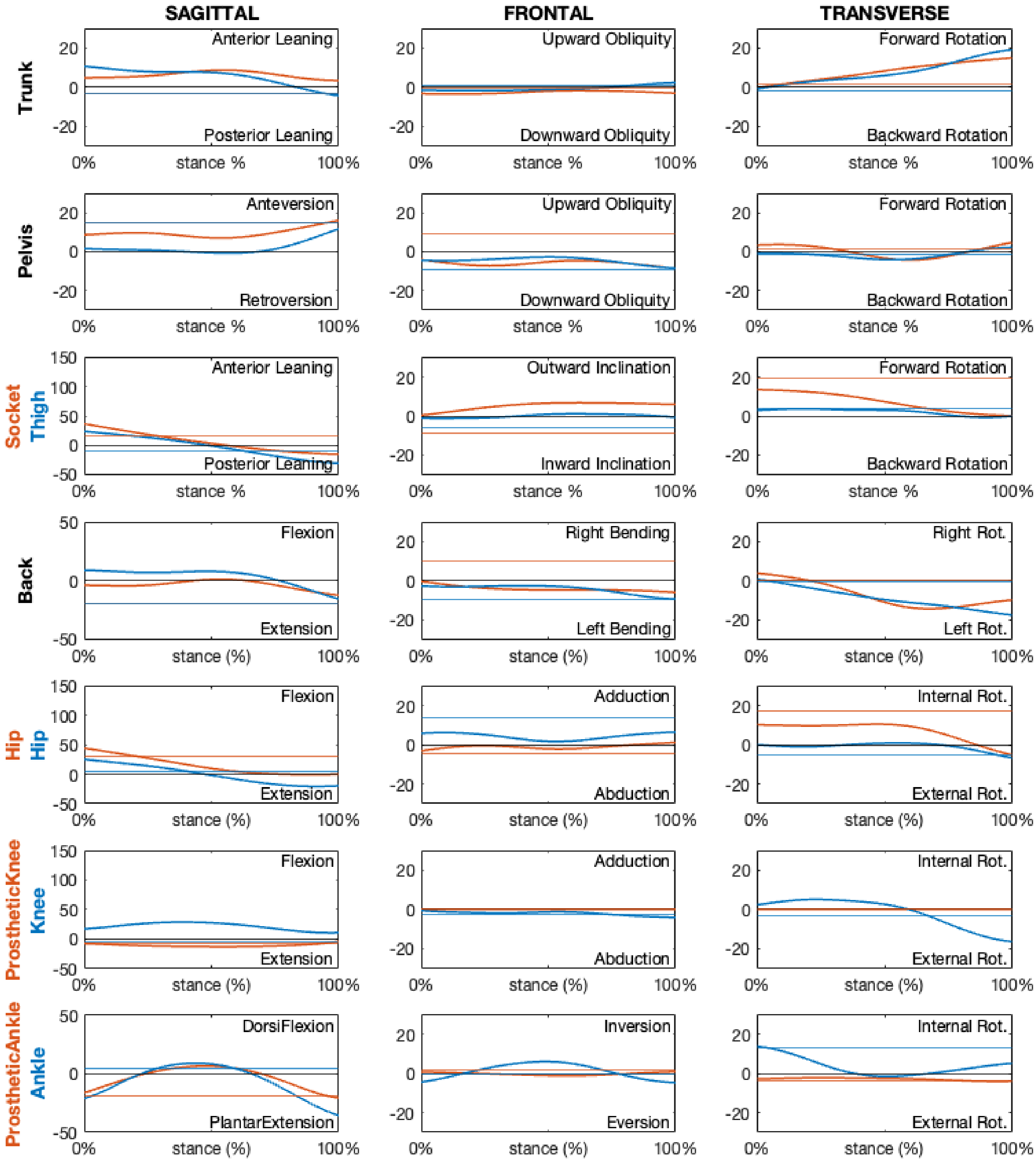

Supplementary Figure 9. Sagittal, frontal and transverse absolute and relative kinematics obtained with the proposed model for a TF amputee during running. Kinematics, reported in blue for non-prosthetic (right) and in dark orange for prosthetic (left) sides, are time normalized over the stance percentage. Horizontal lines show the static kinematics. Vertical lines show the foot-off instants.

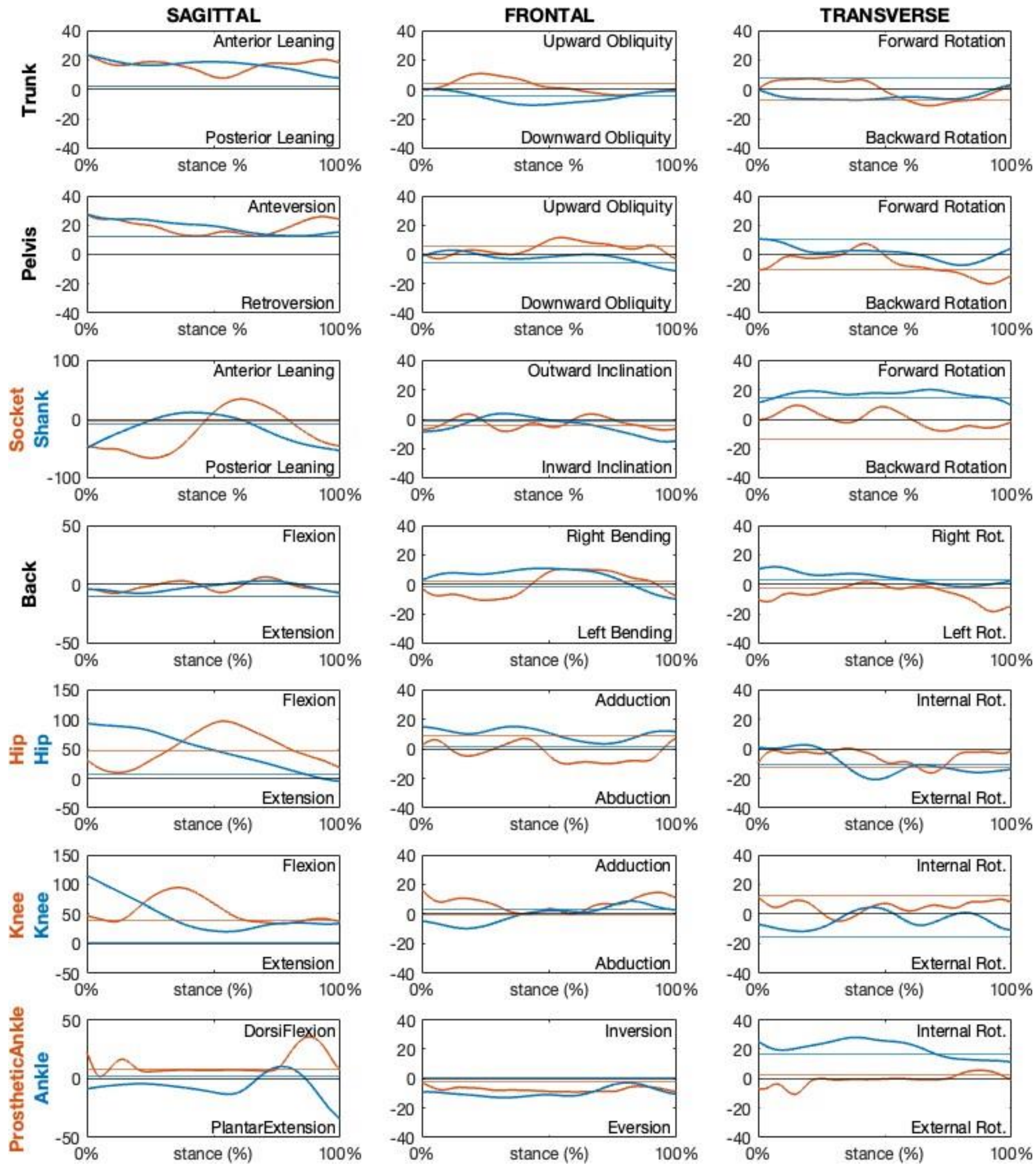

Supplementary Figure 10. Sagittal, frontal and transverse absolute and relative kinematics obtained with the proposed model for a TT amputee during running. Kinematics, reported in blue for non-prosthetic (right) and in dark orange for prosthetic (left) sides, are time normalized over the stance percentage. Horizontal lines show the static kinematics. Vertical lines show the foot-off instants.

7 XML configuration files, MATLAB code to solve the model

7.1 Folder Hierarchy and Files

The codes developed to compute the absolute and relative kinematics of segments and joints as proposed in the present paper rely on the definition of a configuration file given in XML format. The XML config file can be easily built using the *OlympiaConfigurator* application (provided as stand-alone software – see) or manually, editing an empty XML file (file extension will be: *.configuration).

After the installation, the *software* should be stored as follows:

```
> CalibrationObjects
> classes
> files
> functions
v gui
    OlympiaConfigurator.mlapp
    OlympiaMainSW.mlapp
    QuickReportTables.mlapp
    ReportMaker.mlapp
    StaticTrialConstructor.mlapp
    TrialAnalyzer.mlapp
> Imgs
  LICENSE
v Models
    list.models
  > Olympia
> QuickReportTables
  README.md
```

The "Models" folder contains the list.models indexing file, which lists the subfolders within Models. Each subfolder is named after the corresponding model and contains the XML files defining that model. The list.models file is an XML file containing as many entries as the subfolders within Models containing the model definitions: each entry includes the label (i.e., the name of the subfolder) and a short description of the model (optional).

```xml
<?xml version = "1.0" encoding = "UTF-8" ?>
<ModelList>
  <Model label="Olympia" description="Full body model for running and long jumping of paralympic athletes"/>
  <!-- Model label="new model name to be added"
         description="optional field where a short description of the model is given"-->
</ModelList>
```

7.2 Taxonomy and Acquisition management

Given a participant (*subject*) involved in a data collection activity (*session*), this is uniquely identified by the subject identification number and the date of the session. During a session, the subject may be asked to perform tests using different devices: e.g., to compare different prostheses or to walk with and without assistive devices, each associated with specific markers and reference systems. Whenever the configuration of the subject under analysis changes (e.g., testing different alignments of a Running Specific Prosthesis, or testing different Running Prosthetic Feet), a new *configuration* is defined within the same session. Therefore, if a subject is tested with alignment A and alignment B, possibly requiring different marker positioning, these will correspond to configuration A and configuration B, respectively. Similarly, multiple configurations are defined when testing different prosthetic feet.

However, when a new static acquisition is required within the same session, possibly due to one or more markers detached from the subject with their consequent repositioning, this does not correspond to a new configuration but rather to an updated registration of the same configuration.

7.3 Model definition files

The information needed to build the *.configuration file needed to process the static and dynamic according to a specific model are stored in the following XML files:

- <model_name>.markerlist
- <model_name>.coord
- <model_name>.kinematics

Shall the model foresee the use of a marker-equipped pointer to calibrate points relative to a cluster of markers, the *.configuration file contains the information of which pointer should be used and the calibration object is defined in a Wands.calobj XML file (already in the main directory of the *OlympiaConfigurator* software).

The following sections will show their contents and structure. It is worth underlying that, should the Reader want to use the codes provided along with this manuscript to apply any other model, changing the content of the XML files will suffice and allow for computing the kinematics according to any other marker-based model.

The model definition files containing the definitions given in the present manuscript and Supplementary Materials are named:

- Olympia.markerlist
- Olympia.coord
- Olympia.kinematics

7.3.1 Calibration objects XML file

This file contains the definition of the calibration objects that can be used by the *OlympiaConfigurator* to set the reconstruction of pointed markers. The file has the following structure:

```xml
<?xml version = "1.0" encoding = "UTF-8" ?>
<calibration_objects>
   <object_name>
      <TailMarker>mkr1</TailMarker>
      <TipMarker>mkr2</TipMarker>
      <Tail2RealTip>250</Tail2RealTip>
   </object_name>
</calibration_objects>
```

The main node is “calibration_objects”, which contains the calibration object name (e.g., *arrow416* and *object_name*). Each object is defined by:

- *TailMarker*: the closest marker to the wand handle
- *TipMarker*: the most distal marker on the wand, not necessarily coincident with the palpation point
- *Tail2RealTip*: the distance between the TailMarker and the actual tip of the wand used to palpate the points to be reconstructed.

7.3.2 Markerlist XML file

The <model_name>.markerlist file contains the whole marker set having considered all its possible variations to be adapted to different configurations. The file structure has to be as follows:

```xml
<?xml version = "1.0" encoding = "UTF-8" ?>
<MarkerSet label="OLYMPIA">
   <Marker type="T" label="H1" segment="Head" group="General" cluster="1" static="0"
         calibrated="0" opt="0" radius="14" colorR="0.6" colorG="0.7" colorB="0.9"
         visible="1" trajectory="0" />
   <Marker ... />

   <!—- VIRTUAL MARKER DEF -->
   <VirtualMarkerDef label="RCLM" method="centroid" source="RCLAM,RCLPM"/>

   <!—- CALIBRATEDs MARKER DEF -->
   <CalibratedMarkerDef label="RCLM" wand="centroid" source=""/>
</MarkerSet>
```

The main node of the file is *MarkerSet*, whose label reports the name of the model. Each marker record must be defined using the following attributes:

- *type*: Marker type. Possible values are:
    - *A*: Anatomical
    - *T*: Technical
    - *M*: Mechanical
    - *V*: Virtual

      Each virtual marker (*V*) must be complemented by a dedicated definition at the end of the *.markerlist file and within the *MarkerSet* node, through a *VirtualMarkerDef* record. The label of this record must match the label of the corresponding marker node and must include the following attributes:

    - *method*: The computational method used to define the virtual marker (e.g., Bell's method (Bell et al., 1990, 1989)).
    - *source*: The list of points used to compute the virtual marker (e.g., in Bell's method, the markers on the anterior and posterior iliac spines (Bell et al., 1990, 1989).
- *label*: The marker name.
- *segment*: The segment to which the marker belongs.
- *group*: An optional grouping attribute, defined by the user (e.g., *left*, *right*, *general*).
- *cluster* (Boolean):
    - *0* (false, default): The marker is not part of a cluster used for reconstructing other points.
    - *1* (true): The marker belongs to a cluster used to reconstruct other points (Cappello et al., 1997).
- *static* (Boolean):
    - *0* (false, default): Standard marker.
    - *1* (true): The marker is to be removed after the static calibration trial.
- *calibrated* (Boolean):
    - *0* (false, default): Standard marker.
    - *1* (true): The marker is to be calibrated using a calibration wand or regression methods.

      If *calibrated* is set to *1*, the marker will not appear as a *VirtualMarkerDef* even though the *type* is set to *V*. Instead, the *OlympiaConfigurator* will automatically add a *CalibratedMarkerDef* node for each such marker. This node will have the same label as the marker and will include:
        - *wand*: The name of the calibration object selected in the *OlympiaConfigurator*.
        - *source*: This field must be manually filled with the absolute path of the C3D file containing the pointing trial for that marker.
- *opt*: Marker option label:
    - *0* (default): No additional specification.
    - *1*: Optional marker.
    - *2*: Marker used exclusively for transfemoral amputees.
    - *3*: Marker used exclusively for transtibial amputees.
- *radius*, *colorR*, *colorG*, *colorB*, *visible*, *trajectory*: Attributes required to ensure compatibility with C3D visualization software (e.g., Mokka - BTK).

7.3.3 Coord XML file

The `<model_name>.coord` file contains the actual definition of the local coordinate systems given for the adopted model.

The local coordinate systems definition is grouped under the main node *CoordinateSystems*, which may present additional segment grouping (e.g., `General` in the above example) other than the one specified in the *group* attribute of *segment* each node.

```xml
<?xml version="1.0" encoding="UTF-8"?>
<CoordinateSystems>
  <General>
    &lt;Segment label="Head" group="General" points="H1,H2,H3,H4,GB,LAM,RAM,CH" opt="0"
        angleseq="xyz" endpoints="LAM,RAM" inertialalias="Head" comlabel="HDCOM">
      <CoordSys>
        <tag>HD</tag>
        <origin>LAM</origin>
        <origin>RAM</origin>
        <axis1>
          <axis>
            <tail>CH</tail>
            <tip>GB</tip>
          </axis>
        </axis1>
        <temp_axis>
          <axis>
            <tail>GB</tail>
            <tip>origin</tip>
          </axis>
        </temp_axis>
        <seq>yzx</seq>
      </CoordSys>
    &lt;/Segment&gt;
  ...
  </General>
</CoordinateSystems>
```

Each *segment* node is defined by:

- *label*: The name of the segment.
- *group*: Any grouping the user might want to use to help defining the model (e.g., general, left and right).
- *points*: The list of all the points to be used to calculate the coordinate system.
- *opt*: Segment option label:
  - *0* (default): No additional specification.
  - *1*: Optional segment.
  - *2*: Segment used exclusively for transfemoral amputees.
  - *3*: Segment used exclusively for transtibial amputees.
- *angleseq*: The sequence used to decompose the segment's orientation (rotation) matrix with respect to the global reference system.
- *endpoints*: List of markers used to calculate the segment length, which is required for scaling its inertial parameters.
- *inertialalias*: The segment label, as defined in inertial parameter tables (de Leva, 1996).
- *comlabel*: The name of the point associated with the segment’s center of mass.

- *CoordSys*: containing the actual definition of the local coordinate system based on the *points* list, and includes:
    - *tag*: a shorter alternative for the segment label.
    - *origin:* Definition of the coordinate system origin:
        - If only one origin record is provided, the origin corresponds to the specified point label.
        - If multiple origin records are provided, the origin is computed as the centroid of the listed points.
    - *axis1*: Definition of the primary axis. This node can contain one or more *axis* records, each defined by *tail* and *tip* points:
        - If an *axis* is defined by one *tail* and one *tip*, it is computed as the unit vector from *tail* to *tip*.
        - If an *axis* is defined by two *tail* points and one *tip*, it is computed as the unit vector from the midpoint of the two *tail* points to *tip*.
        - If an *axis* is defined by one *tail* and two *tip* points, it is computed as the unit vector from *tail* to the midpoint of the two *tip* points.
        - If *axis1* is defined by two *axis* records, the final axis is computed as the cross product of the first and the second *axis* vectors.
    - *temp_axis*: the definition of a temporary axis, defined as the *axis1*.
    - *seq*: the sequence which the axis definition is provided with.

In the example above, for the head coordinate system, the origin is defined as the midpoint between LAM and RAM. The primary axis (*axis1*) is the unit vector from CH to GB. Having set *seq* to *yzx*, the primary axis is coincident with the *y* axis of the local coordinate system. The temporary axis (*temp_axis*) is the unit vector from GB to the origin. The normal axis to the plane defined by *axis1* and *temp_axis* is the *z* axis of the local coordinate system. The *x* is the last axis to be computed and completes the right-handed orthonormal triad.

7.3.4 Kinematics XML file

The <model_name>.kinematics contains the definition of the joint models and the kinematic chain.

```
<?xml version = "1.0" encoding = "UTF-8" ?>
<kinematics>
  <General>
    <Joint label="Cervical" group="General" prox="Trunk" dist="Head" opt="0"
        angleseq="zxy" type="free" JC="C7" />
    <Joint ... />
  </General>

  <KinematicChain>
    <unaffected_lower group="Left" joints="LeftHip, LeftKnee, LeftAnkle"
              endbody="ForcePlatform" />
    ...
  </KinematicChain>
</kinematics>
```

Each *joint* node is defined by:

- *label*: The name of the joint.

- *group*: Any grouping the user might want to use to help defining the model (e.g., general, left and right).
- *prox*: The label of the proximal segment of the joint.
- *dist*: The label of the distal segment of the joint.
- *opt*: Segment option label:
    - *0* (default): No additional specification.
    - *1*: Optional segment.
    - *2*: Segment used exclusively for transfemoral amputees.
    - *3*: Segment used exclusively for transtibial amputees.
- *angleseq*: The Euler/Cardan sequence used to decompose the joint rotation matrix (relative rotation of the distal segment relative to the proximal segment).
- *type*: The type of mechanical joint associated with the model. This is now used as a description, and it does not add any specification to the kinematics calculation.
- *JC*: The joint center label.

The *KinematicChain* node contains the definition of the kinematic chain associated with the most distal limb. Considering the specific application of the model presented within the present manuscript and its Supplementary Materials, the *KinematicChain* node includes:

- *unaffected_lower*: The kinematic chain associated with an unaffected lower limb.
- *tf_lower*: The kinematic chain associated with a lower limb of a person with a transfemoral amputation.
- *tt_lower*: The kinematic chain associated with a lower limb of a person with a transtibial amputation.

Each of these records must include the following attributes:

- *group*: Any grouping the user might want to use to help defining the model (e.g., general, left and right).
- *joints*: The ordered list of joint labels, from the most proximal to the most distal joint, onto which the external reactions should be projected.
- *endbody*: The source body from which the measured reactions (i.e., ground reaction forces and moments) are measured and projected along the kinematic chain.

7.4 Example of complete configuration file for data processing

The *OlympiaConfigurator* (Supplementary Figure 11) builds the *.configuration file obtaining the needed information from the above-described files and according to the settings chosen by the operator.

Two examples of configuration files are attached to this Supplementary Materials:

- example_left_TF_C_fullbody.configuration: containing the configuration file for the full body model applied on a subject with a transfemoral amputation on the left lower limb.
- example_right_TT_J_lowerbody.configuration: containing the configuration file for the lower body model applied on a subject with a transtibial amputation on the right lower limb.

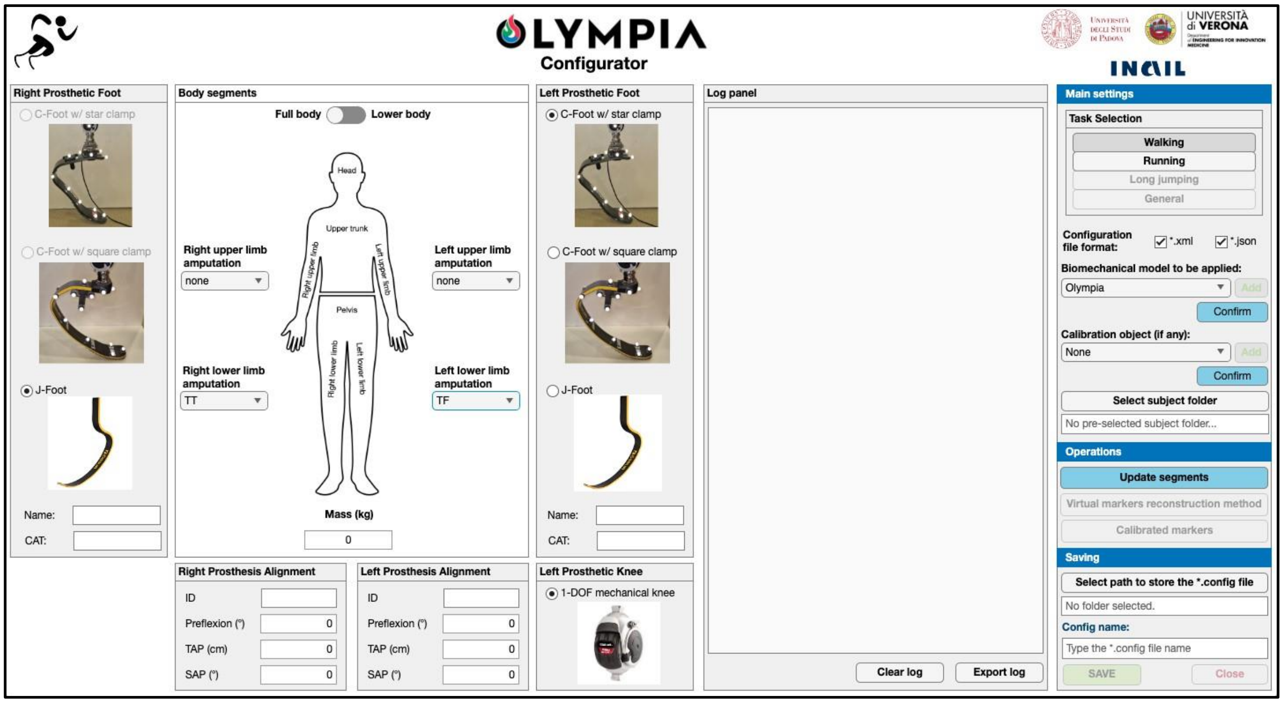

Supplementary Figure 11. The Graphical User Interface of the *OlympiaConfigurator* software.

7.5 MATLAB code

The *OlympiaConfigurator* and the coding to compute the kinematics starting from the C3D files recorded during the data collection sessions are available and commented at:

https://www.doi.org/10.5281/zenodo.15537123

https://github.com/ProOlympia-INAIL-UNIPD/Olympia-MATLAB

7.6 Future update and development

Code maintenance will be ensured, and a Python version of the Olympia Software is currently under development.

References

Bell, A.L., Pedersen, D.R., Brand, R.A., 1990. A comparison of the accuracy of several hip center location prediction methods. J Biomech 23, 617–621.

Bell, Alexander L, Brand, Richard A, Pedersen, Douglas R, Bell, A L, Brand, R A, Pedersen, D R, 1989. Prediction of hip joint center location from external landmarks, Human Movement Science. https://doi.org/10.1016/0021-9290(87)90226-0

Cappello, A., Cappozzo, A., La Palombara, P.F., Lucchetti, L., Leardini, A., 1997. Multiple anatomical landmark calibration for optimal bone pose estimation. 1Human Movement Science 16, 259–274.

Cappozzo, A., Catani, F., Della Croce, U., Leardini, A., 1995. Position and orientation in space of bones during movement: Anatomical frame definition and determination. Clinical Biomechanics 10, 171–178.

de Leva, P., 1996. Adjustments to Zatsiorsky-Seluyanov's segment inertia parameters. J Biomech 29, 1223–1230.

Migliore, G.L., Petrone, N., Hobara, H., Nagahara, R., Miyashiro, K., Costa, G.F., Gri, A., Cutti, A.G., 2021. Innovative alignment of sprinting prostheses for persons with transfemoral amputation: Exploratory study on a gold medal Paralympic athlete. Prosthet Orthot Int 45, 46–53. https://doi.org/10.1177/0309364620946910

Shu, L., Ni, Q., Yang, X., Chen, B., Wang, H., Chen, L., 2020. Comparative study of the tibial tubercle-trochlear groove distance measured in two ways and tibial tubercle-posterior cruciate ligament distance in patients with patellofemoral instability. J Orthop Surg Res 15, 209. https://doi.org/10.1186/s13018-020-01726-2

Wu, G., Siegler, S., Allard, P., Kirtley, C., Leardini, A., Rosenbaum, D., Whittle, M., Lima, D.D., Cristofolini, L., Witte, H., Schmid, O., Stokes, I., 2002. ISB recommendation on definitions of joint coordinate system of various joints for the reporting of human joint motion - part I: ankle, hip, and spine. J Biomech 35, 543–548.
